# XX Disorder of Sex Development is associated with an insertion on chromosome 9 and downregulation of *RSPO1* in dogs (*Canis lupus familiaris*)

**DOI:** 10.1371/journal.pone.0186331

**Published:** 2017-10-20

**Authors:** Vicki N. Meyers-Wallen, Adam R. Boyko, Charles G. Danko, Jennifer K. Grenier, Jason G. Mezey, Jessica J. Hayward, Laura M. Shannon, Chuan Gao, Afrah Shafquat, Edward J. Rice, Shashikant Pujar, Stefanie Eggers, Thomas Ohnesorg, Andrew H. Sinclair

**Affiliations:** 1 Baker Institute for Animal Health, Cornell University, Ithaca, NY, United States of America; 2 Department of Biomedical Sciences, Cornell University, Ithaca, NY, United States of America; 3 Department of Biological Statistics and Computational Biology, Cornell University, Ithaca, NY, United States of America; 4 Department of Genetic Medicine, Weill Cornell Medical College, New York, NY, United States of America; 5 Murdoch Children’s Research Institute, Royal Children's Hospital, Melbourne, VIC, Australia; 6 Department of Paediatrics, University of Melbourne, Melbourne, VIC, Australia; Leibniz Institute on aging - Fritz Lipmann Institute (FLI), GERMANY

## Abstract

Remarkable progress has been achieved in understanding the mechanisms controlling sex determination, yet the cause for many Disorders of Sex Development (DSD) remains unknown. Of particular interest is a rare XX DSD subtype in which individuals are negative for *SRY*, the testis determining factor on the Y chromosome, yet develop testes or ovotestes, and both of these phenotypes occur in the same family. This is a naturally occurring disorder in humans (*Homo sapiens*) and dogs (*C*. *familiaris)*. Phenotypes in the canine XX DSD model are strikingly similar to those of the human XX DSD subtype. The purposes of this study were to identify 1) a variant associated with XX DSD in the canine model and 2) gene expression alterations in canine embryonic gonads that could be informative to causation. Using a genome wide association study (GWAS) and whole genome sequencing (WGS), we identified a variant on *C*. *familiaris* autosome 9 (CFA9) that is associated with XX DSD in the canine model and in affected purebred dogs. This is the first marker identified for inherited canine XX DSD. It lies upstream of *SOX9* within the canine ortholog for the human disorder, which resides on 17q24. Inheritance of this variant indicates that XX DSD is a complex trait in which breed genetic background affects penetrance. Furthermore, the homozygous variant genotype is associated with embryonic lethality in at least one breed. Our analysis of gene expression studies (RNA-seq and PRO-seq) in embryonic gonads at risk of XX DSD from the canine model identified significant *RSPO1* downregulation in comparison to XX controls, without significant upregulation of *SOX9* or other known testis pathway genes. Based on these data, a novel mechanism is proposed in which molecular lesions acting upstream of *RSPO1* induce epigenomic gonadal mosaicism.

## Introduction

At least 50% of human Disorders of Sex Development (DSD) remain unexplained by variants in known genes [1]. Recent reviews have detailed key genes, signaling pathways, and transcriptional networks in the opposing testis and ovary pathways in mammals [1–7]. Key players in the testis pathway include the mammalian Y-linked testis determination gene *SRY* (sex determining region Y), and the vertebrate autosomal testis determination gene *SOX9* (sex determining region Y-box 9). In the ovary pathway, key players include *RSPO1* (R-spondin 1), *WNT4* (Wnt family member 4), *CTNNB1* (catenin beta 1), and T cell/lymphoid enhancer transcription factors (TCF/LEF). Multiple lines of evidence suggest that *SOX9* is a target of both pathways [2]. Although *Sox9* is expressed at low levels in XY and XX gonads at the murine bipotential gonad stage, it is upregulated then maintained by *Fgf9* during a critical period for testis induction [7]. Conversely, ovary pathway components have been identified that suppress *Sox9* transcription. Nevertheless, XX mice with double knockouts of *Rspo1* and *Sox9* develop ovotestes and their XY siblings develop hypoplastic testes [8], indicating that *Sox9* is not essential to testis development. Thus the genetic control of testicular and ovarian development appears complex, and more studies are needed in additional mammals to fully understand the causative mechanisms for DSD.

Two phenotypes recognized in human XX DSD are testicular (T-XX DSD) and ovotesticular (OT-XX DSD). Externally, these individuals have masculinized or ambiguous genitalia [9–10]**.** Variants previously identified in some T-XX DSD patients likely promote the testis pathway through *SOX9* upregulation or ectopic expression of other genes in the *SOX* family, and in some cases are associated with duplications or other copy number variants (CNV) [11–15]. Conversely, *RSPO1* variants that likely disrupt the ovary pathway were identified in a family segregating T-XX DSD [16] and one OT-XX DSD patient [17]. Interestingly, the human homozygous null *RSPO1* variant produced T-XX DSD [16], whereas OT-XX DSD was produced by analogous variants in transgenic mice [18–19]. In human inherited XX DSD, most families segregate either T-XX DSD or OT-XX DSD, not both. However, in a rare subtype, both phenotypes occur in siblings or other related individuals [20–27]. This suggests a causative variant that is identical by descent yet allows variation in phenotypic expression, perhaps due to modifier loci, epigenetic factors, imprinting, or combinations thereof. Causative variants have not been identified in such families. This XX DSD subtype occurs naturally in dogs, where it has been reported in at least 28 dog breeds [28], but the mode of inheritance has not been studied in most. Although CNVs were identified in the *SOX9* coding region [29] and upstream or downstream of *SOX9* [30,31] in sporadic canine cases, none of these has been confirmed as a cause of inherited XX DSD. We proposed that the causative variant for inherited canine XX DSD is likely to be identical by descent, particularly in closely related breeds, such as American and English cocker spaniels [28]. The purposes of this study were to identify a variant associated with XX DSD in the canine model and gene expression alterations in embryonic gonads that could be informative to causation. We identified a variant that is associated with inherited canine XX DSD and found that ovary pathway genes were downregulated in canine embryonic gonads at risk of XX DSD.

## Results

### GWAS identified a CFA9 region associated with XX DSD in the model pedigree

#### The canine XX DSD model pedigree

This pedigree was established in 1982 by crossing a male (ACS1) from an American cocker spaniel (ACS) family segregating XX DSD [32] to normal beagle (BGL) females obtained from a commercial pedigree in which XX DSD has not been identified [33]. Matings of F1 offspring to each other, and F1 backcrosses to ACS1, produced affected mixed breed (ACS/BGL) dogs [33]. No affected F1 females were identified. Only parents of affected dogs were retained as breeding stock at each generation. Additional normal female beagles were later introduced periodically to reduce inbreeding depression. A second American cocker spaniel sire (ACS2) that had produced affected offspring was introduced into the pedigree in 2005. He was bred to females in the pedigree and beagles, and the same breeding strategy as above was used to maintain the model.

All XX DSD in the model pedigree had a female karyotype (78,XX), were *SRY*-negative by PCR assay and had a complete uterus [33–35]. Affected phenotypes included testes (T-XX DSD), ovotestes (OT-XX DSD), and those with an ovotestis and female external genitalia (subclinical XX DSD) with or without ovarian dysfunction [28,33,35,36]. Dogs with a normal male karyotype (78,XY) were unaffected, but were presumed proven carriers after producing affected offspring. Matings of proven carrier males to beagles did not produce affected dogs. Inheritance, based on early breeding experiments, was consistent with sex-limited, autosomal recessive transmission [33]. However, prevalence and severity of XX DSD significantly decreased later as BGL background increased in the pedigree (1982–2005, S1 Fig, S1 and S2 Appendices), indicating that simple Mendelian inheritance alone was insufficient to account for XX DSD expression.

#### GWAS region identified in the XX DSD model pedigree

Twenty-seven dogs were genotyped. Those from the model pedigree were 17 XX DSD, ACS1, ACS2, and 4 females that had produced affected offspring (presumed proven carriers, Table 1). The range of XX DSD phenotypes were mild (1 ovotestis paired with 1 ovary) to severe (bilateral testes). Twelve of the XX DSD were descendants of ACS1, and 5 were descendants of ACS2. Because dogs with female external genitalia in the model may have subclinical XX DSD, 4 BGL females from the commercial vendor were chosen as normal female controls.

**Table 1:** Characteristics of dogs included in GWAS (N = 27).

				Unaffected = 1	Gonad Histology
Breed	Dog ID#	Sire	Dam	XX DSD = 2	XX DSD
BGL	A304	0	0	1	
BGL	A452	0	0	1	
BGL	A888	0	0	1	
BGL	A1050	0	0	1	
ACS1 PC	C205	0	0	XY male	
ACS2 PC	C3428	0	0	XY male	
ACS/BGL PC	C672	C205	BGL	1	
ACS/BGL PC	C871	C734	BGL	1	
ACS/BGL PC	C750	C205	BGL	1	
ACS/BGL PC	C3466	C3428	C3053	1	
ACS/BGL	C709	C205	C333	2	2 ovt>.5t
ACS/BGL	C726	C676	C333	2	2 ovt < .5t
ACS/BGL	C783	C734	C743	2	2 ovt>.5t
ACS/BGL	C795	C734	C750	2	2 ovt < .5t
ACS/BGL	C798	C734	C750	2	2 ovt>.5t
ACS/BGL	C2032	C752	C948	2	2 ovt>.5t
ACS/BGL	C2080	C2005	C854	2	2 t
ACS/BGL	C2085	C2005	C871	2	1 ovt>.5t, 1 ovt < .5t
ACS/BGL	C3006	C1012	C948	2	2 ovt>.5t
ACS/BGL	C3023	C1012	C854	2	2 ovt < .5t
ACS/BGL	C3072	C1012	C871	2	2 ovt < .5t
ACS/BGL	C3120	C1012	C3038	2	1 ovt, 1 ov
ACS/BGL	C3457	C3428	C3052	2	2 ovt < .5t
ACS/BGL	C3468	C3428	C3053	2	2 ovt < .5t
ACS/BGL	C3481	C3428	C3440	2	2 t
ACS/BGL	C3497	C3442	C3466	2	2 ovt < .5t
ACS/BGL	C3549	C3472	C3466	2	2 ovt < .5t

ACS1 and ACS2 are American cocker spaniel (ACS) founder sires of the XX DSD model pedigree. Beagle females (BGL) were obtained from a commercial source. All others are offspring in the XX DSD model pedigree. PC = proven carrier (parent of an XX DSD), ACS/BGL = variable proportions of ACS and BGL genetic background. Gonadal histology types in XX DSD: ovary (ov), ovotestis with less than half testis (ovt < .5t) or greater than half testis (ovt>.5t) and bilateral testes (2 t) (Examples in S2 Fig).

**Fig 1:**
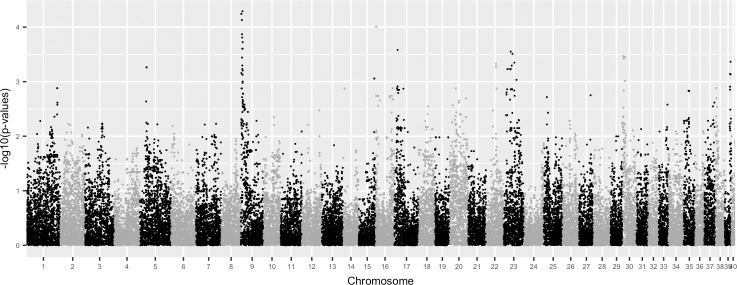
GWAS Manhattan plot for logistic mixed model analysis showing association of XX DSD to CFA9 in the canine model pedigree. The probability of association [-Log_10_ (*P*)] is shown for single nucleotide polymorphisms (SNPs) on chromosomes 1–40. (Quantile-Quantile plot of the p-values is in S3 Fig).

We performed a genome-wide association study (GWAS) of the 27 dogs genotyped on the Affymetrix version 2 canine SNP array (www.broadinstitute.org/mammals/dog/)). A logistic mixed model [37] was then applied to each of the genotypes, to account for breed structure and relatedness of dogs in the pedigree (Fig 1, S1 Table, S3 Fig, S3–S5 Appendices). We then assessed whether the most significant peak of genotypes in the region of CFA9 (*GWAS region*:2605238–6874718 CanFam3.1, genome.ucsc.edu), which included uncorrected p-valuesas low asp <0.0001, indicated suggestive genomic variants for further investigation. Given that an experiment with a sample size of twenty-seven is extremely under-powered when applying a standard GWAS multiple test correction, we used a strategy of applying a multiple test correction after heavy filtering of SNPs to lower the test correction burden, by removing SNPS with missing genotypes, low minor allele frequency SNPs (MAF <0.3), and by thinning every 10^th^ SNP, leaving less than 2,000 SNPs total. After such filtering, we found one SNP in the CFA9 peak region that had an Benjamini-Hochberg (BH) adjusted p-value <0.1, where the next most significant BH adjusted p-value was p-value = 0.297. While SNPs in the CFA9 region were therefore not statistically significant by criteria used in GWAS with samples of thousands of unrelated individuals, given our extremely small sample size, we considered the significance level of SNPs in the CFA9 region to be suggestive enough for further investigation. When considering the 32 SNPs spanning the peak of the CFA9 region, this 4.27MB region overlaps the canine ortholog for the upstream regulatory region of human *SOX9* [38,39] and the canine ortholog for human XX DSD (Online Mendelian Inheritance in Man, OMIM 278850, hg38 chr17:69100001–76800000). The GWAS region does not include the canine *SOX9* coding region, the canine ortholog of the mouse testis-specific enhancer of *Sox9* (TESCO) [40], or the canine ortholog of human *RevSex* [13]. This region is in LD with SNPs spanning the CNVR1 region that was previously identified in controls and some cases of canine XX DSD [30], but does not overlap this region.

#### Post GWAS SNP genotyping and MLPA studies

To narrow the 4.27MB CFA9 GWAS region to a smaller area around the causative allele, genotypes were assembled by chromosomal locus to identify SNPs common to ACS1 and ACS2, and thus indicate an affected haplotype. Two haplotype blocks were identified by homozygosity mapping in which the alternate SNP alleles were homozygous in ACS1 and ACS2 and the reference SNP alleles were homozygous in 4 female controls. A custom canine genomic tiling array (Roche Diagnostic Corp., see Further associated variant discovery, Methods) was used for SNP genotyping to further narrow down the candidate interval within the combined haplotypes totaling 2MB (2604935–3349954 and 5922063–7019389; CanFam3). Two XX DSD (C3023, C3481), and ACS2 from the model pedigree and 2 female controls (A304, A888, Table 1) were genotyped with this array in the 2MB region, identifying 5 SNP haplotype blocks (*Regions 1–5*) that were shared by ACS2 and affected dogs, but not by controls. SNPs within these haplotype blocks were validated by Sanger sequencing of PCR products. When the CanFam3 assembly became available post-analysis, we converted the 5 SNP haplotype blocks to CanFam3 coordinates (LiftOver function, UCSC Genome browser), but found no change in their relative positions on CFA9. Further investigation of these blocks by homozygosity mapping was based upon the principle that regions inherited from the ancient domestic dog population tend to be transmitted whole, as they are shorter than intra-breed haplotype blocks that range from 0.5–1 MB [41]. Thus, affected purebred dogs can have alternate alleles that are identical‐by‐descent at markers located near a disease locus [41]. *Region 3* had the highest proportion of homozygosity for the alternate, rather than the reference, SNP allele in 28 affected dogs in the study pedigree, and in 26 unrelated affected dogs of 21 breeds. In contrast, homozygosity of alternate alleles was shared only within the study pedigree in *Regions 2*, *4*, *5*, and only in the study pedigree and ACS and ECS breeds in *Region 1*. Two variant SNP alleles (6397782 and 6398082; CanFam3) in *Region 3* haplotype block segregated with XX DSD in a total of 20 and 12 breeds respectively, including the Afghan hound, the most ancient breed tested. However, these two SNP alleles were also identified in female controls. Taken together, the data suggested that these two SNPs were breed-associated, but could be markers for a nearby variant in a shared haplotype block from the ancient domestic dog population that contains *Region 3*.

As CNVs have been associated with human XX DSD, particularly those mapping to the *RevSex* region within the *SOX9* regulatory region [13], we hypothesized that alternate SNPs in the *Region 3* haplotype might indicate the presence of a CNV. Therefore, we used Multiplex Ligation-dependent Probe Amplification (MLPA) to screen for CNVs in the *Region 3* SNP haplotype block and the canine *RevSex* ortholog (S2 and S3 Tables, see Methods: MLPA for CNV detection). Because no probes in the canine *RevSex* ortholog had been included in the GWAS genomic tiling array for SNP genotyping, this region was also SNP genotyped by Sanger sequencing of PCR products (see Methods, SNP genotyping). No variations unique to XX DSD dogs were identified by MLPA or SNP genotyping in the *Region 3* haplotype block or canine *RevSex* ortholog. These results in the canine *RevSex* ortholog are comparable to another study in which no CNVs uniquely associated with canine XX DSD were identified [30], and differ from studies in which CNVs including *SOX9* or upstream of *SOX9* were identified in sporadic canine XX DSD cases [29,31].

### Whole genome sequencing identified an XX DSD associated region in the GWAS region

**Fig 2:**
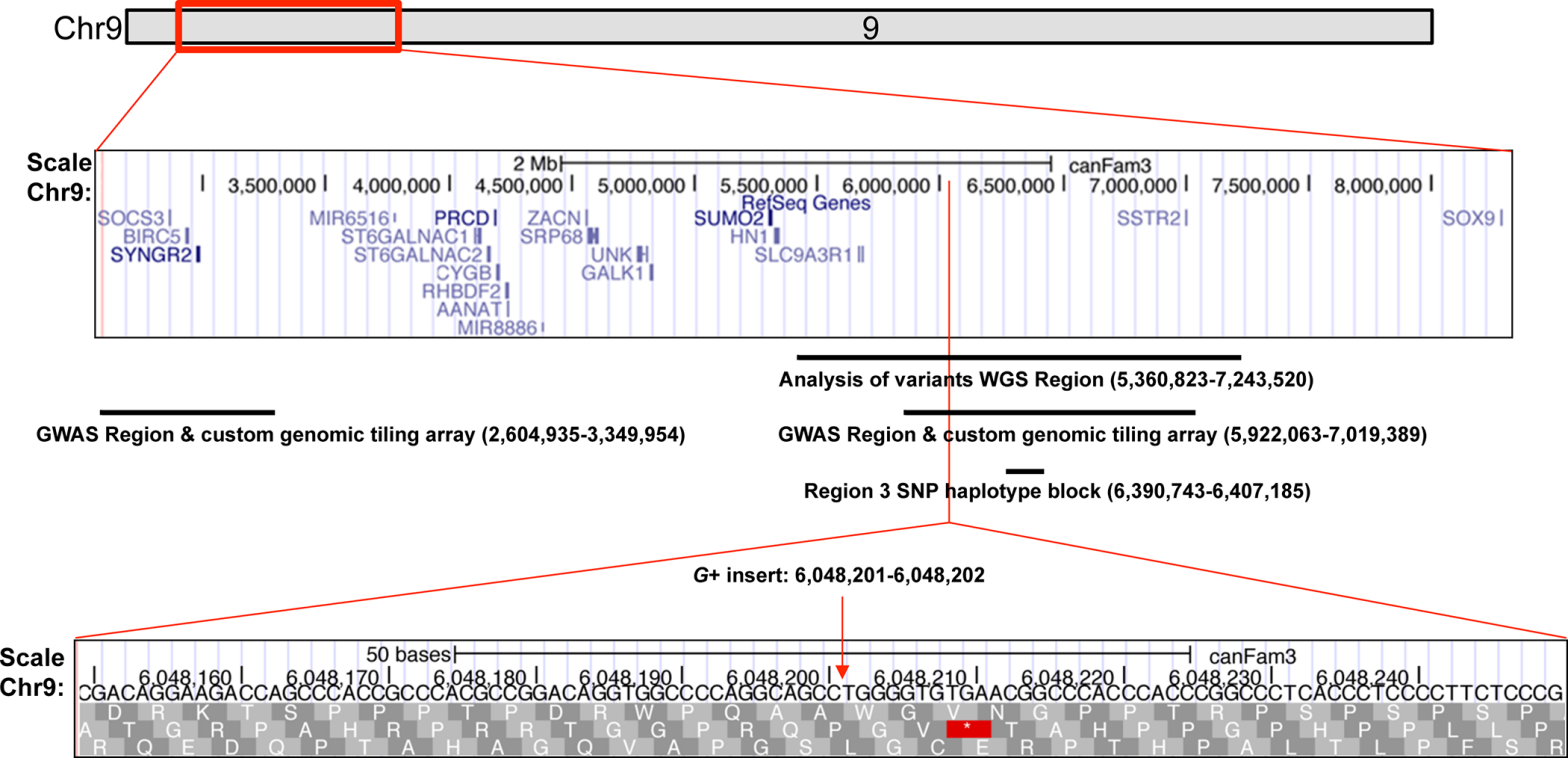
Genomic regions on CFA9 associated with canine XX DSD, indicated by method of identification. The top ideogram of CFA9 (UCSC browser, CanFam 3/3.1) shows the entire span in which regions associated with canine XX DSD were found in this study. Red lines indicate magnified portions of this span, which are screen shots from the browser. Horizontal bars approximate the location of each region identified. Text below the bars indicates the method by which a region was identified, and its specific coordinates. The bottom screen shot shows the location of the *G+* insert (arrow) associated with canine XX DSD.

Since a causal variant was not directly identified by GWAS, and there were an insufficient number of recombinant individuals in the model pedigree for further fine mapping, we elected to use whole genome sequencing (WGS) to identify additional markers associated with XX DSD. We selected 4 dogs for WGS. Based upon our previous findings of an autosomal recessive inheritance pattern of a major causative locus [33], the 2 affected dogs (OT-XX DSD) were expected to be homozygous for the causative variant. Both were from the model, but one (C783) was a descendant of ACS1, and the other (C3549) was a descendant of ACS2 (Table 1). The third dog was a female (C750) produced by ACS1 and a female beagle, and she had produced XX DSD offspring (Table 1). Therefore she was predicted to be an obligate heterozygote carrier. The normal female control (A168), predicted to be homozygous wild type, was a female from the Persistent Mullerian Duct Syndrome pedigree, which does not segregate XX DSD [42]. Sequencing depth after mapping reads was: 6.38 (C783), 6.32 (C3549), 7.91 (C750) and 7.30 (A168). Of 18,600 SNP/Indel alleles genome-wide matching the predicted segregation pattern in the 4 dogs, 244 are located in a 1.9MB segment of CFA9 (*WGS region*:5360823–7243520; CanFam3.1), an area of >16 fold enrichment for variant alleles that lies within the *GWAS region* (Fig 2, data in S4 Table). Results indicated variants in the WGS region were associated with XX DSD in the model, but these individuals were too closely related to be useful in differentiating between causative and linked variants. Therefore, to determine which of the 244 WGS variants were associated with XX DSD, we next tested affected dogs that were unrelated to the model.

#### Fine mapping by analysis of WGS variants in XX DSD and control purebred pet dogs identified a CFA9 insertion associated with the condition

Based upon a hypothesis that XX DSD prevalence in several dog breeds indicates most breeds inherited the same risk factors from an ancestral domestic dog population [41], we predicted that the major causative genetic defect and associated variants are located within such a canine ancestral segment that, characteristically, has short linkage disequilibrium (LD), and due to small size (~100-500kb), this segment would tend to be inherited intact. We used a custom designed canine SNP array with a maximal limit of 84 allele-specific probes. Variants were selected from the 244 loci on CFA9 identified by WGS. High priority was assigned to unannotated variants with allele frequencies less than 8% (AF<0.08) in a canine WGS database [43], which excluded approximately half having an alternate AF >0.2. Additional priority was assigned to those in which the reference allele was conserved, with lower priority assigned to variants in repeats annotated in the UCSC Genome browser (CanFam3). The final selection included 75 test loci from the *WGS region* (CFA9:5360823–7243520, CanFam3.1, Fig 2) and 9 published SNPs for quality control (CFA9:5369117–6097313, listed in S5 Table).

The array was used to genotype DNA from 3 groups, totaling 189 purebred pet dogs unrelated to the XX DSD model pedigree (Table 2). The first group, *Affected Pets*, was expected to be enriched for the causal variant found in the model pedigree because XX DSD in these dogs was confirmed by the PI (M-W), using the same diagnostic criteria as in the model pedigree. The *Control Pets 1* group contained purebred dogs having normal female external genitalia that were breed-matched to the Affected Pets. These were expected to be normal females, unidentified carriers, or subclinical XX DSD dogs. The latter two were expected to have the causative allele. The *Control Pets 2* group contained purebred dogs having normal female external genitalia from breeds in which XX DSD has not been reported. The majority of these were expected to be normal females, with a minority of unidentified carriers or subclinical XX DSD dogs having the causative allele. Based upon our hypothesis of a common causative variant for XX DSD in purebred dogs, we predicted the allele frequency of an XX DSD associated variant common to several breeds would be highest in the Affected Pets group, lower in the Control Pets 1 group, and lowest in the Control Pets 2 (AF Affected Pets group > AF Control Pets group 1 > AF Control Pets group 2). The results were compatible with this hypothesis (Table 2). Genotypes were obtained from 188 samples tested in the custom designed canine SNP array. After filtering 8 probes with low call rate (<70%), the remaining 76 loci had an average call rate of 96%, and 100% concordance in duplicate samples. Variant genotypes sorted by identification number, breed and group were tested for XX DSD association. One variant, a guanine insertion (*G+*) at CFA9: 6048201–6048202 (Table 2, Fig 2, rs852549625) was identified as significantly associated with XX DSD (Chi-squared test, P = 1.1e-6 using dominant model of association, Bonferroni threshold 6.6e-4). This variant was not annotated in the canine WGS database [43] or canine genome (CanFam3) at the time it was selected for screening by the array.

**Table 2:** Purebred pet dog groups genotyped with the custom array of selected WGS variants to identify those that are common to affected dogs of several breeds.

	Individual	XX DSD in	Dogs	Breeds	*G+* allele	% Genotypes at CFA9:6,048,201			
Group	phenotypes	breeds tested	tested	tested	frequency	*G+G+*	*G+/-*	*(-/-)*	*CT SNP*
Affected	XX DSD	reported	N = 63	N = 22	0.405	3	75	21	2
Pets									
Control	no XX DSD	reported	N = 65	N = 20	0.292	9	42	48	2
Pets 1									
Control	no XX DSD	not reported	N = 60	N = 20	0.175	5	25	65	5
Pets 2									

Genotypes at the CFA9:6048201 insertion locus are: homozygous for the guanine insertion (*G+G+*), heterozygous for the insertion (*G+/-*), wild type (-/-), or heterozygous *C/T* (CT SNP). At this locus, the guanine insertion allele frequency was significantly different between the Affected Pets and Control Pets 1 and 2 (P = 8.99 x 10^−3^, P = 2.9 E-07, respectively, Chi-squared test), and between Control Pets 1 and 2 (P = 4.31 x 10^−4^, Chi-squared test). By group, insertion allele frequencies were consistent with the hypothesis prediction for an XX DSD associated variant that is inherited from an ancestral population, being highest in affected dogs, intermediate in those from the same breeds as affected dogs, and lowest in breeds in which XX DSD has not been reported (AF = 0.405 > AF = 0.292 > AF = 0.175). The majority of Affected Pets (75%) were *G+/-* genotype, including American cocker spaniels, of which 11 were *G+/-* and 1 was *G+G+* genotype. A novel CT SNP was identified at this locus in 1 dog in each of 5 breeds (S6 Table). Altogether, the guanine insertion was identified in at least 1 dog in 32 breeds, including 11 breeds in Control Pets 2. Although XX DSD has not been reported in these 11 breeds, 4 of these are related to breeds in which XX DSD has been reported [44]. The guanine insertion was not found in 9 breeds in Control Pets 2 (S6 Table). These breeds are in clades in which XX DSD has not been reported [44].

Genotyping at the insertion locus (Fig 2) was repeated by Sanger sequencing of PCR products from all dogs tested in the custom SNP array. Electropherogram analysis for each dog (S6 Table) confirmed the array results, indicating that the XX DSD associated allele was a single guanine insertion at this locus, and that no flanking alleles associated with XX DSD were present. No deletions or other insertions uniquely associated with XX DSD in the model pedigree were identified by Sanger sequencing of contiguous PCR products within CFA9:6046484–6048494. Furthermore, no CNVs were identified by Droplet Digital PCR (ddPCR) near the 6048201 locus, or in the *SOX9* coding region in XX DSD from the model pedigree (probes listed in S7 Table). Taken together, results indicate that the 6048201guanine insertion is a marker for inherited canine XX DSD.

The guanine insertion is located in *BTBD17* intron 1, which is orthologous to human chr17:74360240–74361728 (hg38,—strand, LiftOver function, genome.ucsc.edu). The guanine insertion also lies within a CpG island (CpG26, CanFam3.1, genome.ucsc.edu ). *KIF19* and an unannotated protein (F1PLR5) are nearby. Many potential transcription factor binding sites are present within 125 bp of CFA9:6048201. The guanine insertion introduces potential PAX sites, whereas none are here in the reference genome, and adds potential C2H2 ZF and bHLH sites to those already present (listed in S8 Table).

#### Genotype results at the 6048201 locus indicate that breed background affects penetrance and support a complex mode of inheritance for canine XX DSD

In contrast to XX DSD purebred pet dogs, in which most were heterozygous for the insertion (75% *G+/-*, Table 2), most XX DSD in the model pedigree were homozygous for the insertion (83% *G+G+*, Table 3). Both founder sires (ACS1, ACS2) were *G+G+* and all BGL females bred into the model pedigree were wild type (*-/-*). The full range of phenotypes was found in dogs with *G+G+* genotype in the model pedigree, which had varying proportions of ACS and BGL background. Interestingly, 5 ACS/BGL breeding stock with female external genitalia that had produced affected offspring were *G+G+* genotype (Table 3). Gonads from these dogs were removed at 2.5–9 years of age and no seminiferous tubules were identified by histology. Presently there is not a method to definitively determine whether these were unaffected females or the mildest XX DSD phenotype (subclinical XX DSD) that was not ascertained by gonadal histology.

**Table 3:** Most XX DSD in the model pedigree (83%) were homozygous for the guanine insertion (*G+G+*) at CFA9: 6048201.

				Genotype
Animal			Gonad	at CFA9
Number	Sire	Dam	Histology	6048201
C743	C676	C671	2 ov	*G+G+*
C989	C752	C743	2 ov	*G+G+*
C3052	C1012	C871	2 ov	*G+G+*
C3440	C3428	C3064	2 ov	*G+G+*
C3441	C3428	C3064	2 ov	*G+G+*
C3567	C3428	C3535	1 ovt, 1 ov	*G+G+*
C3542	C3472	C3441	1 ovt, 1 ov	*G+G+*
C730	C205	C672	1 ovt, 1 ov	*G+G+*
C796	C734	C750	1 ovt, 1 ov	*G+/-*
C2021	C734	C871	1 ovt, 1 ov	*G+G+*
C2026	C752	C989	1 ovt, 1 ov	*G+/-*
C3120	C1012	C3038	1 ovt, 1 ov	*G+/-*
C2085	C2005	C871	1 ovt >.5t, 1 ovt < .5t	*G+G+*
C726	C676	C333	2 ovt < .5t	*G+G+*
C795	C734	C750	2 ovt < .5t	*G+G+*
C930	C734	C786	2 ovt < .5t	*G+G+*
C964	C752	C854	2 ovt < .5t	*G+G+*
C3103	C2005	C3052	2 ovt < .5t	*G+G+*
C3104	C2005	C3052	2 ovt < .5t	*G+G+*
C3022	C2005	C948	2 ovt < .5t	*G+G+*
C3023	C1012	C854	2 ovt < .5t	*G+/-*
C3072	C1012	C871	2 ovt < .5t	*G+G+*
C3457	C3428	C3052	2 ovt < .5t	*G+G+*
C3468	C3428	C3053	2 ovt < .5t	*G+G+*
C3497	C3442	C3466	2 ovt < .5t	*G+G+*
C3670	C3428	C3596	2 ovt < .5t	*G+/-*
C3549	C3472	C3466	2 ovt < .5t	*G+G+*
C709	C205	C333	2 ovt >.5t	*G+G+*
C798	C734	C750	2 ovt >.5t	*G+G+*
C2032	C752	C948	2 ovt >.5t	*G+G+*
C3006	C1012	C948	2 ovt >.5t	*G+G+*
C783	C734	C743	2 ovt >.5t	*G+G+*
C2080	C2005	C854	2 t	*G+G+*
C3481	C3428	C3440	2 t	*G+G+*
C3582	C3428	C3466	2 t	*G+G+*

All XX DSD and their parents were genotyped by Sanger sequencing of PCR products and electropherogram analysis. These dogs vary in proportion of American cocker spaniel and beagle breeds (ACS/BGL) and are sorted by gonadal histology results. Gonadal histology types: ovary (ov), ovotestis (ovt) with less than half testis (ovt < .5t) or greater than half testis (ovt>.5t) and bilateral testes (2 t). (Examples in S2 Fig) The full range of affected phenotypes was identified in dogs with *G+G+* genotypes. Milder phenotypes (1 ovt to 2 ovt < .5t) were identified in those with *G+/-* genotypes (shaded). Listed at the top are 5 ACS/BGL breeding stock that produced XX DSD offspring. These were *G+G+* genotype, and are likely subclinical XX DSD, but seminiferous tubules were not identified in gonadal histology.

#### The homozygous insertion is associated with embryonic lethality in combination with German shorthaired pointer (GSHP) genetic background

**Fig 3:**
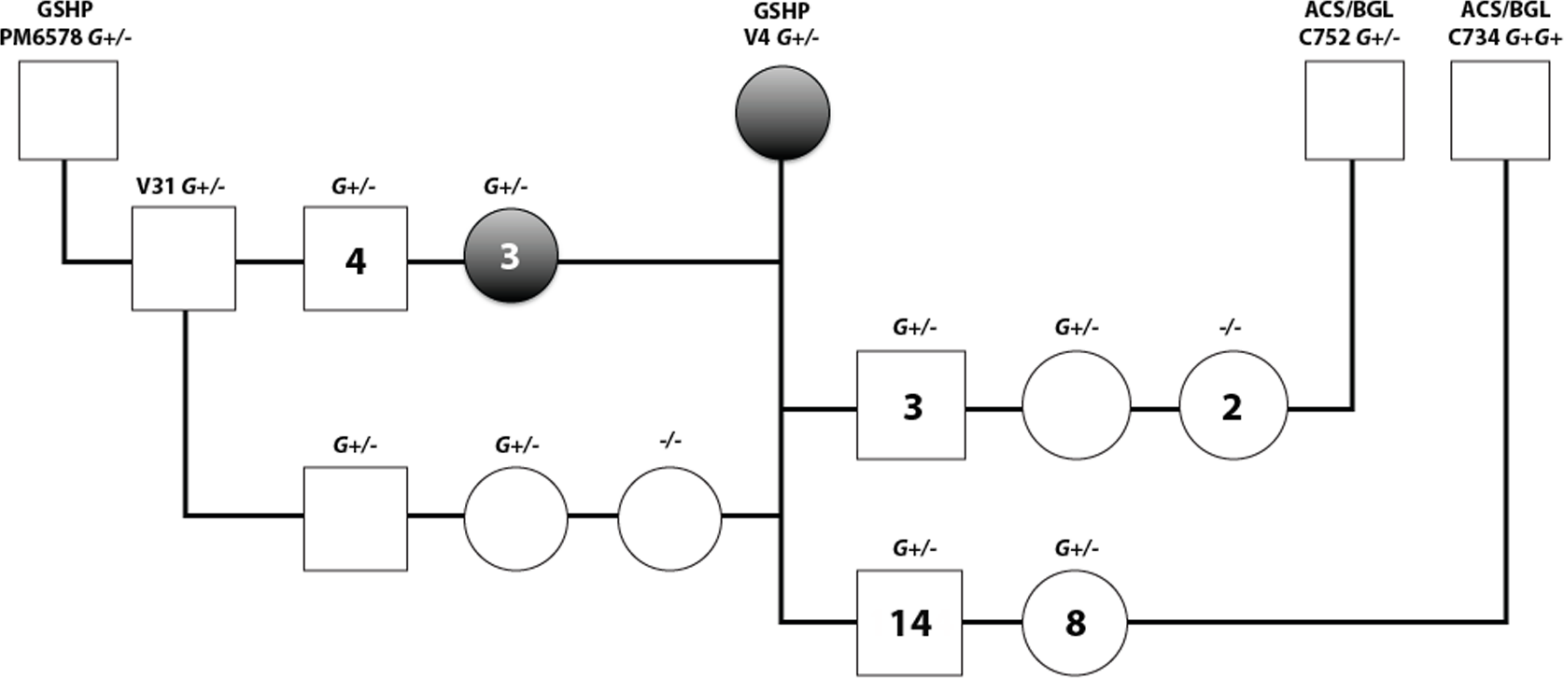
Embryonic lethality in association with the homozygous insertion at CFA9:6048201 was identified in breeding experiments. At left are crosses between V4, a fertile GSHP XX DSD and GSHP males (PM6578, V31). At right are crosses between V4 and males from the XX DSD model pedigree (C752, C734), which vary in proportion of American cocker spaniel and beagle genetic background (ACS/BGL). Genotypes at the insertion locus (CFA9:6048201) are homozygous (*G+G+*), heterozygous (*G+/-*), or wild type (*-/-*). Females are indicated by open circles, XX DSD by filled circles, and XY males by squares. Each symbol represents one dog, except those containing a number, which indicates the number of dogs represented by that symbol. None of the offspring had the *G+G+* genotype. Based on simple Mendelian inheritance, the expected number of *-/-*, *G+/-* and *G+G+* offspring from the *G+/-* sires and dams is 4.25, 8.5, and 4.25, respectively (N = 17). This is significantly different from the numbers observed (*x*^2^ = 8.1776, df = 2, Chi-squared test is significant at p<0.025 since 8.1776>7.38). Similarly, the expected number of *G+/-* and *G+G+* offspring from a *G+/-* dam and *G+G+* sire (V4 x C734) is 11 and 11, respectively (N = 22). This is a highly significant difference from the numbers observed (*x*^2^ = 20.04, calculations include continuity correction for df = 1 [45]; Chi-squared test is significant at p<0.005 since 20.04>7.88). Since all offspring of C734 (*G+G+*) must have received the *G+* allele from him, only those receiving the wild type allele from the GSHP V4 dam (*G+/-*) were born. Together, these results indicated that the *G+G+* genotype, combined with ≥50% GSHP background, is associated with embryonic lethality.

Because genotyping results at the 6048201 locus indicated that the *G+G+* genotype was uncommon in XX DSD purebred pet dogs (3% *G+G+*, Table 2), but common in mixed breed XX DSD in the model pedigree (83% *G+G+*, Table 3), we hypothesized this genotype was deleterious in purebred dogs. Therefore, we genotyped pedigrees from purebred and crossbred breeding experiments in which the probability of *G+G+* offspring varied from 0.25–0.5 (Fig 3). All offspring were born alive and birth weights within the same litter were similar. None of the offspring were *G+G+* genotype, including crossbred offspring sired by males from the model pedigree that had previously produced *G+G+* and *G+/-* XX DSD offspring (sires C734 and C752, Table 3). These results indicated that embryonic lethality was associated with the *G+G+* genotype in the GSHP genetic background (Fig 3).

### Gene expression in embryonic gonads from the canine XX DSD model and controls

#### RNA-seq identified *RSPO1* downregulation but not *SOX9* upregulation in gonads from the XX DSD model

The second purpose of this study was to identify gene expression alterations in embryonic gonads from the model pedigree that could be informative to XX DSD causation. As these are unique and rare samples, we chose RNA-seq to measure all expressed genes, as well as known genes in the testis and ovary pathways. Because embryonic gonad pairs at the ages tested had insufficient mass for both RNA-seq and histology, the guanine insertion at CFA9:6048201 was used as a marker in genomic DNA to identify embryos at risk of XX DSD from the model pedigree (Table 4).

**Table 4:** Embryonic gonad pairs for RNA-seq were collected at d34-44 from embryos at risk of XX DSD, XY littermate controls, and XX and XY age-matched controls.

A.	Test	Gestational	Animal	6048201		B.	Control	Gestational	Animal	
	group	age	number	Genotype	Litter#		group	age	number	Litter#
	XX d35	d35	C3509	*G+/G+*	194		XX d34 pool	d34	A1056	165
		d35	C3510	*G+/G+*	194			d34	A1057	165
		d35	C3511	*G+/G+*	194			d34	A1060	165
		d35	C3525	*G+/-*	197			d34	A1063	166
		d35	C3571	*G+/G+*	208			d34	A1067	166
		d35	C3572	*G+/-*	208			d34	A1068	166
		d35	C3573	*G+/-*	208		XY d34 pool	d34	A1055	165
		d35	C3575	*G+/-*	208			d34	A1058	165
		d35	C3616	*G+/-*	217			d34	A1059	165
	XY d35	d35	C3574	*G+/G+*	208			d34	A1061	165
		d35	C3617	*G+/-*	217			d34	A1064	166
		d35	C3618	*G+/G+*	217			d34	A1065	166
		d35	C3619	*G+/G+*	217			d34	A1066	166
	XX d37-39	d37	C3512	*G+/G+*	195		XX d37-39	d37	A1097	170
		d37	C3515	*G+/G+*	195			d37	A1098	170
		d38	C3694	*G+/G+*	230			d37	A1101	170
		d38	C3696	*G+/G+*	230			d39	A1086	169
		d39	C3624	*G+/G+*	219			d39	A1088	169
		d39	C3630	*G+/G+*	220			d39	A1089	169
		d39	C3632	*G+/-*	220		XY d37-39	d37	A1096	170
		d39	C3633	*G+/-*	220			d37	A1099	170
		d39	C3636	*G+/G+*	221			d37	A1100	170
		d39	C3642	*G+/G+*	221			d39	A1091	169
	XY d37-39	d37	C3516	*G+/G+*	195			d39	A1094	169
		d38	C3691	*G+/G+*	230			d39	A1095	169
		d39	C3626	*G+/G+*	219		XX d42-44	d44	A1104	171
		d39	C3627	*G+/G+*	220			d44	A1107	171
		d39	C3637	*G+/G+*	221			d44	A1110	171
		d39	C3638	*G+/G+*	221		XY d42-44	d44	A1106	171
	XX d42-44	d42	C3517	*G+/G+*	196			d44	A1108	171
		d43	C3684	*G+/G+*	229			d44	A1109	171
		d43	C3688	*G+/G+*	229					
		d44	C3659	*G+/G+*	225					
	XY d42-44	d42	C3518	*G+/-*	196					
		d42	C3522	*G+/-*	196					

(A) Embryos at risk of XX DSD chosen from the model pedigree were *SRY-*negative (XX) and homozygous (*G*+/*G*+) or heterozygous (*G*+/-) for the guanine insertion associated with XX DSD. XY littermate controls were *SRY*-positive and homozygous or heterozygous at the insertion locus. (B) Age-matched controls were *SRY*-negative (XX) or *SRY-*positive (XY) embryos produced from wild type (-/-) beagle parents (see Methods).

Gene expression was compared between XX DSD gonads at risk and XX age-matched controls for both the d37-39 and d42-44 age groups. The list of genes with significant expression differences found by cuffdiff v2 was further filtered for minimum expression level in at least one condition and minimum fold-change between conditions to establish a ‘stringent’ set of differentially expressed genes (stringent-DE genes) consisting of 271 genes at d37-39 and 625 genes at d42-44 (combined 754 genes; see Methods). A majority of stringent-DE genes (539) were downregulated in XX DSD gonads in comparison to XX controls. Of these, over 500 were located on chromosomes 1, 5, 6, 9, 13, 20 and 26 (data in S4 Fig). For all chromosomes, the region with the greatest number of downregulated genes was CFA20:50-60M, which contains transcription factor E-2 alpha (*TCF3)*, follistatin-related protein3 (*FSTL3)*, and Anti-Mullerian hormone (*AMH*), also known as Mullerian Inhibiting Substance. On CFA9, the greatest number of downregulated genes (N = 13) was located between 1-10MB, which includes the GWAS and WGS regions. The stringent-DE gene list contained a total of 38 annotated genes on CFA9 (CanFam3), primarily downregulated in XX DSD gonads at risk in comparison to XX age-matched controls. Only four of the stringent-DE genes on CFA9 were significantly upregulated in XX DSD gonads at d42-44, but these have no reported role in gonadal sex determination (ENSCAFG00000004922, ENSCAFG00000017563, ENSCAFG00000017668/*TUBD1*, ENSCAFG00000025142). Expression of 3 annotated genes nearest the insertion (*BTBD17*, *KIF19* and F1PLR5) was very low, with Fragments Per Kilobase of transcript per Million mapped reads (FPKM) being <<1 in all groups. No expression in this region was identified for polyA transcripts expressed in adult ovary (CUFF.38126.1, CUFF.38126.2, CanFam3, genome.ucsc.edu ). To investigate novel splice forms and transcripts within and near the *GWAS* and *WGS regions* (CFA9:5-9MB), a similar RNA-seq analysis was run that was guided by, but not limited to, the CanFam3 Ensembl transcriptome. No novel transcripts with differential expression between XX DSD and XX controls were found in this interval.

**Fig 4:**
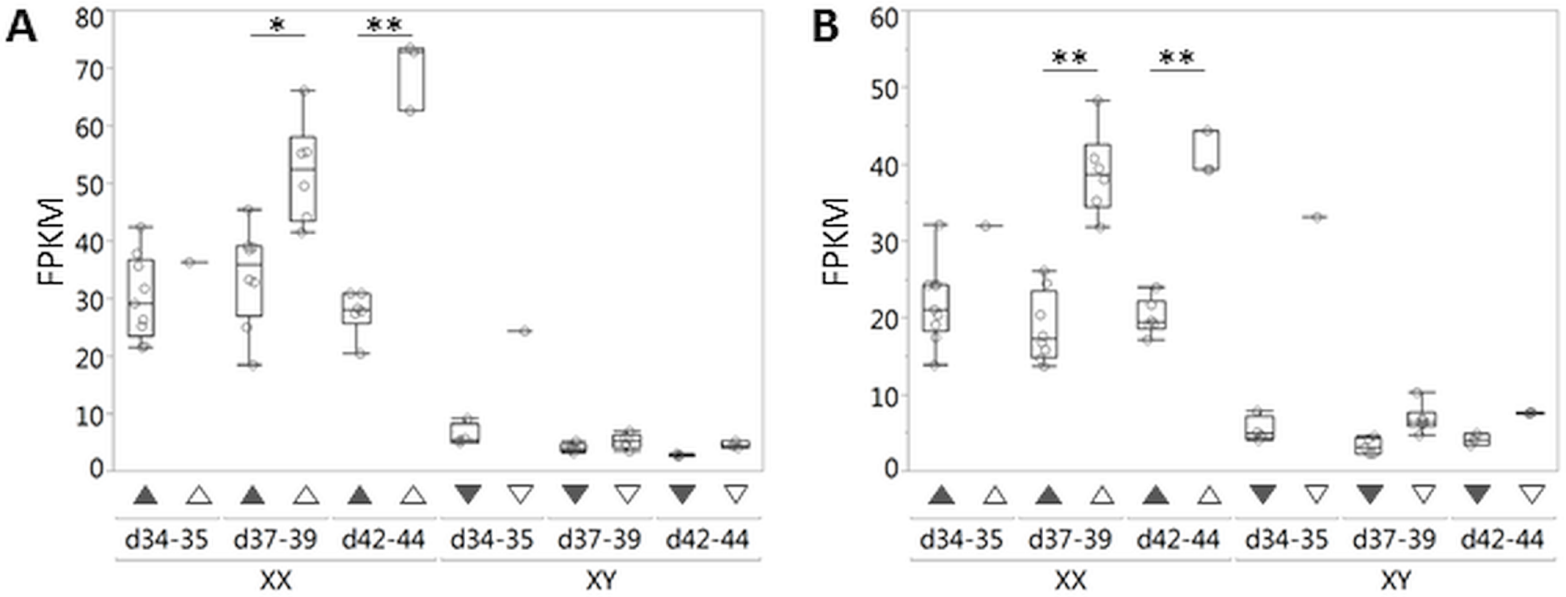
*RSPO1* and *WNT4* expression in canine embryonic gonads was measured by RNA-seq. (A) *RSP01* and (B) *WNT4* expression were lower in XX DSD gonads at risk (filled triangles) compared to those of XX age-matched controls (open triangles) at d37-39 and d42-44. Neither *RSP01* nor *WNT4* expression in XX DSD gonads at risk or those of XX age-matched controls was as low as those of XY littermate controls (filled inverted triangles) or XY age-matched controls (open inverted triangles). Each open circle represents RNA from one embryonic gonad pair, except for d34, which represents RNA from a gonad pool. Differential expression was statistically tested for XX DSD gonads compared to XX controls at d37-39 and d42-44 (* p-value<0.005; ** p-value ≤ 0.00005). FPKM is Fragments Per Kilobase of transcript per Million mapped reads.

**Fig 5:**
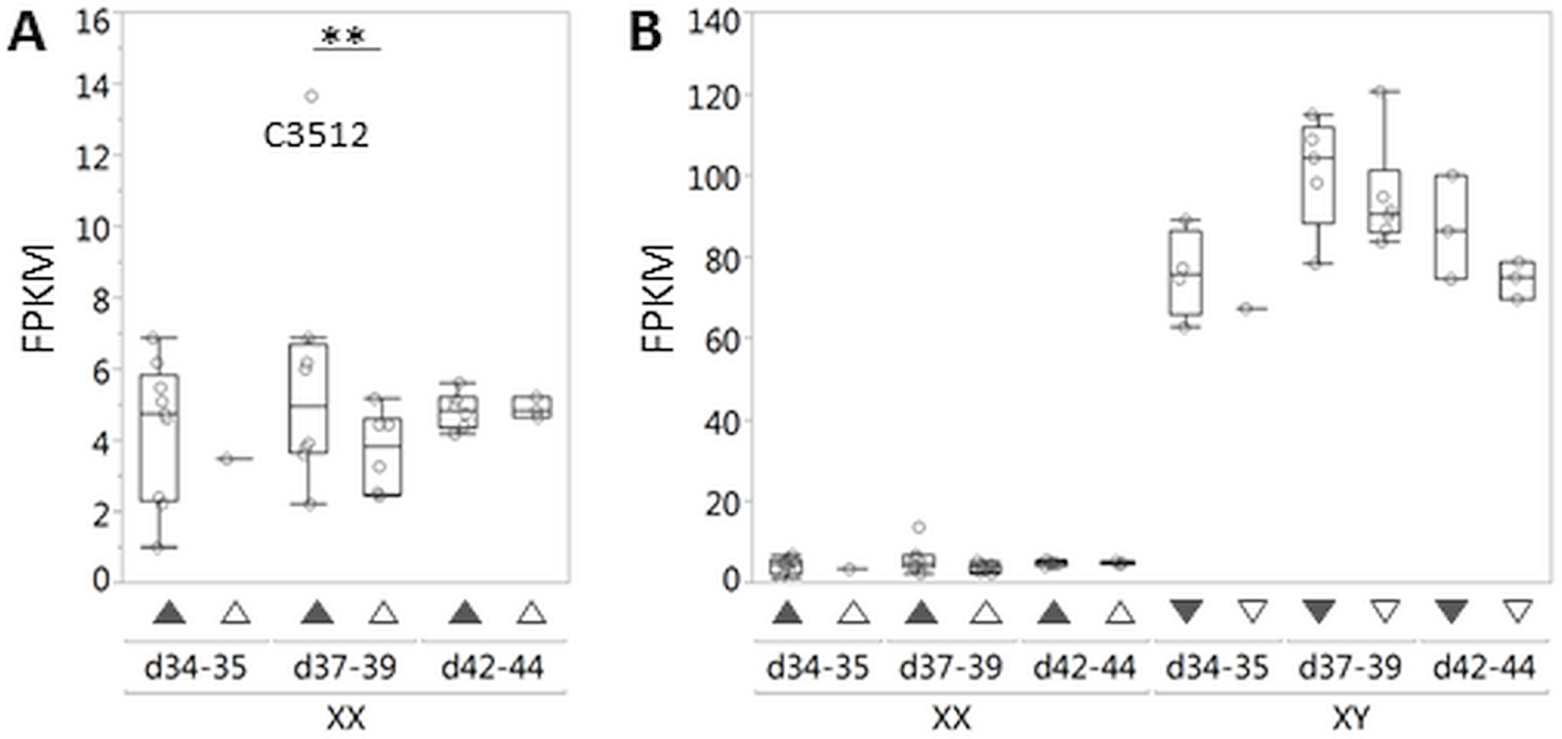
*SOX9* expression in canine embryonic gonads was measured by RNA-seq. (A) At d37-39, *SOX9* expression was significantly greater in XX DSD gonads at risk (filled triangles) compared to XX age-matched control gonads (open triangles), but <2 fold and primarily due to one sample (C3512). Each open circle represents RNA from one embryonic gonad pair, except for d34, which represents RNA from a gonad pool. Differential expression was statistically tested for XX DSD gonads compared to XX age-matched controls at d37-39 and d42-44 (** p-value ≤ 0.00005). (B) At all ages tested, *SOX9* expression in XY littermate (filled inverted triangles) and XY age-matched control gonads (open inverted triangles) was much greater than in XX DSD gonads at risk (filled triangles) and XX age-matched controls (open triangles). Each open circle represents RNA from one embryonic gonad pair, except for d34, which represents RNA from a gonad pool. FPKM is Fragments Per Kilobase of transcript per Million mapped reads.

In general, ovary pathway genes were expressed at lower levels in XX DSD gonads at risk in comparison to XX controls in both age groups (Table 5). In particular, *RSPO1* and *WNT4* expression were lower in XX DSD gonads compared to XX controls (Fig 4). Interestingly, expression was lower by >2 fold for *RNF43* at d42-44 (Table 5), a negative regulator of the WNT/CTNNB1 pathway [46]. The major finding in ovary pathway genes was that *RSPO1* is significantly downregulated at d42-44 using the stringent criteria of >2 fold difference. While a general downregulation of other genes in the ovary pathway is present, these did not meet the stringent criterion of >2 fold difference. Similarly, testis pathway gene expression was higher in XX DSD than XX control gonads, but none met the stringent criterion of >2 fold difference (Table 5). Notably, *SOX9* expression in XX DSD gonads at risk was considerably lower than in XY controls (Fig 5), and no difference in *DMRT1*, *SOX3*, *SOX8*, or *SOX10* expression was detected between XX DSD gonads at risk and female controls. In summary, the major difference between XX DSD gonads at risk and XX gonads was *RSPO1* downregulation at d42-44, without upregulation of known testis pathway genes.

**Table 5:** Ovary and testis pathway gene expression was measured by RNA-seq.

			p_value	p_value
ENSCAFG number	Gene symbol		d37-39	d42-44
		OVARY PATDWAY		
		Statistically significant at both ages		
ENSCAFG00000005204	*CTNNB1*	HIGHER in XX DSD by <2-fold	5.1E-03	5.0E-05
ENSCAFG00000008353	*SFRP2*	HIGHER in XX DSD by <2-fold	5.0E-05	5.0E-05
ENSCAFG00000013324	*LRP6*	HIGHER in XX DSD by <<2-fold	9.0E-04	8.5E-04
ENSCAFG00000003280	*RSPO1*	LOWER in XX DSD & by >2-fold d42-44	4.2E-03	5.0E-05
ENSCAFG00000014661	*WNT4*	LOWER in XX DSD by ~2-fold	5.0E-05	5.0E-05
ENSCAFG00000032086	*CBX2*	LOWER in XX DSD by <2-fold	6.0E-04	5.0E-05
ENSCAFG00000011973	*ZNRF3*	LOWER in XX DSD by <2-fold	5.5E-03	5.0E-05
ENSCAFG00000007585	*FOXL2*	LOWER in XX DSD by <2-fold	2.1E-02	5.0E-05
ENSCAFG00000011339	*LHX9*	LOWER in XX DSD by <<2-fold	6.0E-04	5.0E-05
ENSCAFG00000011236	*AXIN2*	LOWER in XX DSD by <<2-fold	7.9E-03	4.50E-02
	* *	Significant at d37-39 age group only		
ENSCAFG00000016200	*TCF12*	HIGHER in XX DSD by <<2-fold	6.8E-03	
	* *	Significant at d42-44 age group only		
ENSCAFG00000010188	*LGR4*	HIGHER in XX DSD by <<2-fold		9.5E-03
ENSCAFG00000017526	*RNF43*	LOWER in XX DSD by >2-fold		2.0E-03
ENSCAFG00000000990	*TCF7*	LOWER in XX DSD by <2-fold		5.0E-05
ENSCAFG00000010871	*LRP5*	LOWER in XX DSD by <2-fold		3.0E-04
	* *	Not significant at either age		
ENSCAFG00000011252	*LEF1*	(p-value > 0.05)		
		TESTIS PATHWAY		
	* *	Statistically significant at both ages		
Anti-Mullerian hormone	*AMH*	LOWER in XX DSD by ~2-fold	5.0E-05	5.0E-05
	* *	Significant at d37-39 age group only		
ENSCAFG00000004374	*SOX9*	HIGHER in XX DSD by <<2-fold	5.0E-05	
	* *	Significant at d42-44 age group only		
ENSCAFG00000029890	*FGF9*	HIGHER in XX DSD by <2-fold		5.0E-05
		BIPOTENTIAL		
	* *	Significant at d42-44 age group only		
ENSCAFG00000023086	*SF1*	LOWER in XX DSD by <2-fold		5.0E-05
	* *	Not significant at either age		
ENSCAFG00000007426	*WT1*	(p-value > 0.05)		

Differences in expression between XX DSD gonads at risk and XX age-matched controls were tested at gestational age groups d37-39 and d42-44. An overall trend for downregulation in the ovary pathway was identified in XX DSD gonads at risk. Based on stringent criteria (>2 fold difference), *RSPO1* expression was significantly lower at d42-44 in XX DSD gonads at risk, but expression of known testis pathway genes was not significantly elevated. Genes listed under BIPOTENTIAL are expressed in both the ovary and testis during development.

#### PRO-seq confirmed RNA-seq results

**Fig 6:**
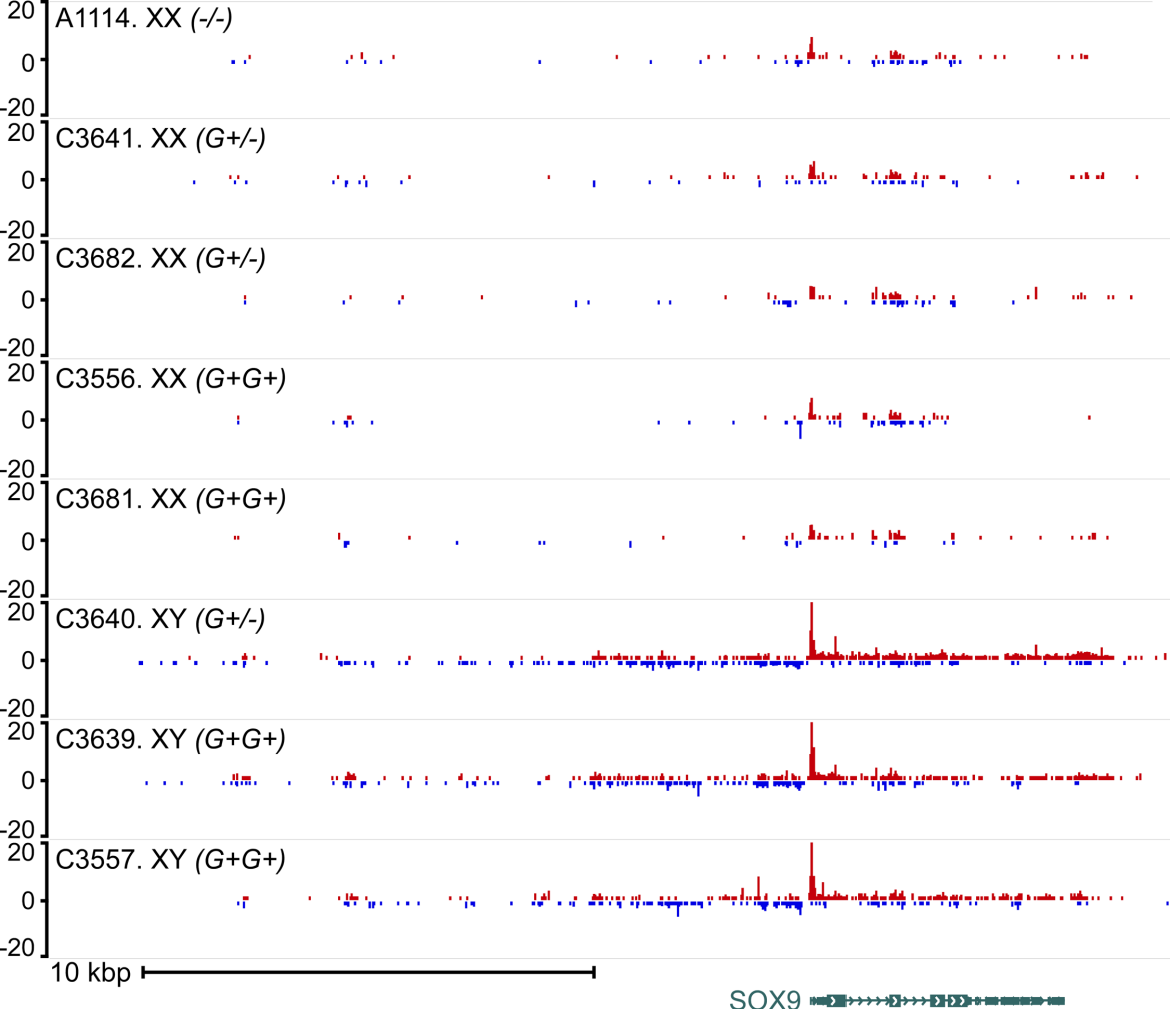
Genome browser view of the raw PRO-seq signal near the *SOX9* gene in d39 gonads. *SOX9* expression was not significantly elevated in d39 XX DSD gonads at risk compared to those of XX age-matched controls. The *SOX9* signal is highest in XY littermate control gonads (C3640, C3639, C3557) and much lower in the age-matched XX control (A1114) and XX DSD gonads at risk (C3641, C3682, C3556, C3681). For each sample, histograms show reads aligned to the plus strand (top, red) and minus strand (bottom, blue). Listed at left are embryo ID number, group (XX/XY) and genotype at the insertion locus: wild type (*-/-*), homozygous (*G+G+*), and heterozygous (*G+/-*) for the guanine insertion associated with XX DSD. RefSeq gene annotation is shown below the plot (CanFam3.1).

PRO-seq provides insights into gene regulation by measuring the location and density of active RNA polymerase genome-wide. It produces a quantitative picture of gene transcription at a single time point [47], and identifies the direction of gene transcription (+/- strand). Therefore, we used PRO-seq as a second assessment of embryonic gonadal gene expression. Embryos (d39) were produced from the model pedigree and a beagle control litter (Table 6). Results confirmed those of RNA-seq at d39 (Table 5). High signal intensity near *RSPO1* was found in XX age-matched control gonads, lower signals were found in the XX DSD gonads at risk and lowest signals in XY littermate controls. However, there were insufficient sample numbers to statistically test these differences. High signal intensity near *SOX9* was found in XY littermate control gonads, with low signals in XX DSD gonads at risk and XX age-matched controls (Fig 6). Signals were not detected near *BTBD17*, *KIF19* and F1PLR5.

**Table 6:** Canine embryonic gonad pairs collected for PRO-seq.

	Animal	6048201		Gestational
Group	number	genotype	Litter#	age
XX age-matched control	A1114	*(-/-)*	172	d39
XX DSD at risk	C3641	*G+/-*	221	d39
XX DSD at risk	C3682	*G+/-*	228	d39
XX DSD at risk	C3556	*G+G+*	204	d39
XX DSD at risk	C3681	*G+G+*	228	d39
XY littermate control	C3640	*G+/-*	204	d39
XY littermate control	C3639	*G+G+*	221	d39
XY littermate control	C3557	*G+G+*	221	d39

The female beagle control (XX age-matched control) was *SRY*-negative and wild type (-/-) at the insertion locus. Male controls (XY littermate control) were *SRY*-positive. Embryos at risk of XX DSD (XX DSD at risk) were *SRY*-negative (XX) and homozygous (*G+/G+)* or heterozygous (*G+/-*) for the insertion associated with XX DSD (CFA9:6048201).

## Discussion

In this type of inherited XX DSD in humans and dogs, *SRY*-negative individuals have a normal female karyotype but develop testes or ovotestes, and both of these phenotypes occur in the same family. Affected individuals develop ambiguous or masculinized genitalia and are often infertile. The etiology of this type of inherited XX DSD in humans and dogs is unknown.

### The variant associated with canine XX DSD

The first purpose of this study was to identify a variant associated with XX DSD in the canine model. The guanine insertion associated with XX DSD is located in the GWAS and WGS regions (CFA9:6048201) and within the canine ortholog for this type of XX DSD in humans (OMIM 278850). This region is not located in the CFA9 region (CanFam3.1) in which CNVs were identified in controls or cases of canine XX DSD in other studies [29–31]. However, assuming that the CanFam3.1 assembly is accurate, this variant is in LD with, but does not overlap CNVR1 [30]. Taken together, it suggests that this CFA9 region contains elements regulating gonadal sex determination and possibly some elements essential to embryonic viability.

This is the first variant to be associated with inherited XX DSD in the canine model and in unrelated purebred dogs. As a marker of the affected haplotype, it can be used to predict disease risk. Further studies are warranted to determine whether it has a role in pathogenesis. It is likely to be an ancestral variant in the domestic dog population, as we have shown it is associated with XX DSD in several breeds. Genotyping at the insertion locus showed that the majority of affected dogs in the model pedigree were homozygous for the XX DSD associated insertion (83% *G+G+*, Table 3). These dogs varied in proportion of ACS and BGL genetic background. In contrast, homozygotes were uncommon in affected purebred pet dogs (3% *G+G+*, Table 2), as most were heterozygous at this locus (75% *G+/-*, Table 2). This small percentage of *G+G+* genotypes in XX DSD purebred dogs could result from infertility in affected heterozygotes, leaving only a small number of fertile dams carrying the *G+* allele. However, breeding experiments demonstrated that a *G+G+* genotype in combination with the GSHP background was associated with embryonic lethality (Fig 3). It is likely that this lethal effect occurs in other breeds, since the *G+G+* genotype was uncommon in all purebred pets tested, but this requires further investigation.

We note that in previous studies, we performed a whole genome linkage analysis screen of the canine XX DSD model pedigree using microsatellite markers on CanFam1 [48]. A CFA29 region was linked to XX DSD, but fine mapping revealed no other linked markers. We now consider the CFA29 linkage to be a false positive result due to CanFam1 assembly errors. In particular, *SOX9* and the *SOX9* linked markers used in the linkage study were located on CFA18 in CanFam1. In subsequent assemblies (CanFam2 and 3) *SOX9* is located on CFA9, while the *SOX9* linked markers used in the linkage study are located on CFA18. Therefore retrospectively, there were no *SOX9* linked markers in the linkage study.

### Gene expression in canine embryonic gonads

The second purpose of this study was to identify gene expression alterations in canine embryonic gonads that could be informative to XX DSD causation. RNA-seq results indicate that *RSPO1* is very significantly downregulated in gonads at risk of XX DSD at d42-44 (Table 5, Fig 4). Thus the XX DSD genetic defect is likely acting upstream of *RSPO1*. The general downregulation of the ovary pathway would explain ovarian dysfunction observed in OT-XX DSD and subclinical XX DSD phenotypes. It is more difficult to explain how this induces testis development (T-XX DSD and OT-XX DSD). Although *SOX9* expression at d37-39 was significantly greater in XX DSD gonads at risk compared to those of XX age-matched controls, it did not meet the stringent criteria of >2 fold difference, and was primarily due to high expression in one embryo (Fig 5). Furthermore, *SOX9* expression in all XX DSD gonads at risk, including the one outlier, was far less than expression in both XY control groups. No evidence of significant *SOX3*, *SOX8*, *SOX10*, or *DMRT1* upregulation was detected between XX DSD gonads at risk and female controls. PRO-seq confirmed that *SOX9* is not upregulated at d39 in XX DSD gonads at risk (Fig 6). These data suggest that testis induction in XX DSD occurs even though testis pathway gene expression is far below that of XY males. An alternate explanation is that testis pathway gene expression may be undetectable by RNA-seq in whole embryonic mosaic gonads unless testis induction has occurred in a majority of the gonad. In that case, single cell RNA-seq or novel methods may be required to measure expression differences due to gonadal mosaicism. Finally, we cannot rule out that differences between XX affected and XX age-matched control gonads may be attributable to a portion of the ACS genetic background that does not include the XX DSD mutation. However, it should be possible to test this question when a causative mutation, and its function in producing XX DSD, are confirmed.

Unfortunately, gonads were too small to be analyzed by RNA-seq or PRO-seq at d30-32, the age in which *SOX9* is first expressed in normal canine testes [49]. However, it is unlikely that *SOX9* is upregulated in XX DSD gonads earlier than the ages tested, since delayed testis development and delayed testicular AMH secretion are features of the canine XX DSD model. The first seminiferous tubules and AMH secretion were identified at d34 in normal testes, but not until d40 in XX DSD gonads [36]. Therefore, it is reasonable to expect that *SOX9* upregulation would have been detected in XX DSD gonads at the ages tested (d35-44), as it was detected in XY control gonads. Testing at earlier ages in normal canine embryos may be informative for expression of genes near the insertion, such as *BTBD17* and *KIF19*. While no significant gonadal expression was detected for these genes at d34-44 in controls, either could be expressed earlier, perhaps before *RSPO1* expression normally begins in ovaries. Functionality of potential transcription factor binding sites introduced by the *G+* insertion near these genes could be tested by in vitro luciferase assays [50]. Briefly, luciferase reporter constructs containing a potential site and two controls, one containing the native canine sequence, the other being an empty plasmid, could be prepared such that each site is located at an appropriate distance from a minimal promoter. After transfection of each of these together with the appropriate transcription factor expression plasmids, reporter gene expression is measured by chemiluminescent response and compared to those of controls.

In addition to affecting expression of nearby genes, it is possible that the guanine insertion we identified modulates gene expression over a larger DNA segment (CFA9:1-10MB). The insertion lies within intron 1 of *BTBD17*, about which little is known, except that it contains a POZ domain. These domains mediate homomeric or heteromeric dimerization [51] and repress transcription by recruiting components of histone deacetylase corepressor complexes [52]. POZ domains can also interact with HMG proteins in transcriptional complexes [53]. Thus *BTBD17* may interact with HMG proteins such as *SRY*, *SOX9* and *TCF/LEF* transcription factors, which modulate gene transcription in the testis and ovary pathways [54–57]. For example, recent studies found that Klhl31, which contains a POZ domain, is a modulator of beta catenin-dependent Wnt signaling, which balances embryonic myoblast proliferation and differentiation [58]. Finally, proteins containing POZ domains can create chromatin insulator complexes that permit or suppress transcription over large DNA segments [59].

The CpG island location of the guanine insertion may also be relevant to long-range DNA effects. CpG islands are rare unless there is selective pressure for retention [60]. Promoter regions are often associated with CpG islands, most of which contain unmethylated cytosines that are available for protein interactions. When a CpG island contains a methylated cytosine that is not under positive selection for retention, the methylated cytosine spontaneously deaminates over time [61], thereby changing the cytosine (C) to a thymine (T). It is unknown whether the cytosine at the CG site introduced by the guanine insertion is methylated or unmethylated. However, our finding of a CT polymorphism at this locus in 5 dogs (Table 2) could indicate that the C reference allele is methylated in some dogs. Therefore, the G insertion following this methylated C could produce a methylated CG site, and potentially change the methylation pattern within the CpG island. Future experiments in the canine model, such as Enhanced Reduced Representation Bisulfite Sequencing [62] or methylation pyrosequencing [63], could investigate methylation at the CFA9:6048201 site in embryonic gonadal DNA from XX DSD and controls. Methylation of cytosines in a CpG island can reduce transcription [64], reduce binding of transcription factors to a promoter [65], change chromatin state by recruiting methyl-CpG-binding proteins [65], or change allele-specific expression of imprinted genes [66,67]. For example, tissue-specific DNA methylation differences between CpG islands at the non-5’ end of the *KIF19* coding sequence have been identified in neural vs. non neural human tissues [68]. In addition, a mouse *KIF19* ortholog is associated with ovarian development and folliculogenesis [69], and female infertility is reported in *Kif19a* knockout mice [70]. Further examination of this knockout could determine whether testis or ovotestis development is a factor in the female infertility. Similarly, a *Btbd17* knockout and serial targeted alterations within the noncoding ortholog may be useful to identify sequence essential to mouse gonadal development.

### Canine XX DSD is likely inherited as a complex trait

Taken together, genotyping results at the XX DSD associated insertion locus and segregation of genotypes with the phenotypes support the hypothesis that inherited canine XX DSD is a complex trait in which penetrance is modulated by breed genetic background. First, 83% of XX DSD in the mixed breed (ACS/BGL) model pedigree were *G+G+* genotype, and few dogs with *G+/-* genotypes were affected (Table 3). Embryonic lethality was not associated with either genotype in the model pedigree. In contrast, 75% of XX DSD purebred pet dogs had the *G+/-* genotype (Table 2), including 11/12 ACS XX DSD that were unrelated to the model pedigree. Experimental breedings showed that the *G+G+* genotype with GSHP breed background was associated with embryonic lethality (Fig 3). These results suggest that the XX DSD genetic defect alters epigenomic signals over large areas of DNA, which could account for the downregulation observed in many genes in the CFA9:1-10MB region. One of these, *LLGL2*, lies 1MB upstream of the guanine insertion locus and was downregulated in XX DSD gonads at risk compared to XX controls. Murine knockouts of *Llgl2* have abnormal placental growth, embryonic growth retardation and increased neonatal mortality [71]. However, no dogs with the analogous genotype (*G+G+*) were born in breeding experiments (Fig 3), which suggests a different mechanism for embryonic lethality in these dogs. In the context of the model pedigree, genotyping and phenotyping results suggest that factors in the BGL genome may suppress phenotypic expression of the XX DSD genetic defect that is transmitted from the ACS. In that case, the BGL genome would allow survival of *G+G+* genotype embryos. Furthermore, as the proportion of BGL genome increased in pedigree members, the XX DSD phenotype would be expected to decrease from testis to ovotestis, and even to ovary if sufficient BGL genome was present to fully suppress penetrance. However, further studies are needed to understand the breed related factors that modulate XX DSD penetrance and embryonic lethality.

#### An epigenomic model for gonadal mosaicism

An epigenomic model for gonadal mosaicism in the canine XX DSD model could account for the wide range of XX DSD phenotypes observed in related individuals. Phenotypically, gonads observed in the canine XX DSD model are phenocopies of those resulting from sex chromosome mosaicism, which are composed of XX and XY clones developing as a mosaic of ovary and testis. The proportion of ovary or testis depends upon the proportion of XX and XY clones. Similarly, we propose a model of gonadal mosaicism in which the canine XX DSD genetic defect disrupts epigenomic mechanisms that control cell commitment to the testis or ovary pathway. Epigenomic mosaicism has been described in mice having variable expression at the agouti locus. This causes mosaic coat colors in individuals and highly variable expression between littermates according to parent of origin [72]. A similar process is proposed for XX DSD in the canine model. The canine XX DSD associated insertion is located within a putative CpG promoter. These sites have the potential to affect gene regulation over large areas of DNA and can be sensitive to genomic background and methylation patterns that differ by parent of origin. For example, a recent study showed that multiple methylation sites imprinted by parent of origin influenced gene expression over a 1.9MB region on human chromosome 15 [73]. Their population analyses of differentially methylated regions predict that loss of imprinting at such sites could cause congenital defects that have incomplete penetrance or variable expression. This may be applicable to XX DSD, as differences in methylation pattern and chromatin marks were found in a *RevSex* duplication shared by an XX DSD patient, the XY father, and unaffected paternal grandmother [74]. The epigenomic mosaic model we propose predicts that epigenomic differences in canine XX DSD lead to varying degrees of *RSPO1* downregulation between gonadal clones. In some clones, *RSPO1* downregulation is insufficient to prevent cell commitment to the ovary pathway. In others, it is so severe that cells commit to the testis pathway. Thus a mosaic pattern of ovarian and testicular clones would develop within the same gonad. This mechanism would also account for the wide range of gonadal phenotypes observed in affected individuals from the same family, as well as differences in penetrance observed with changes in canine genomic background. Further studies are needed, since this would be a novel mechanism for inherited canine XX DSD, and possibly for human families segregating this XX DSD subtype.

#### The canine XX DSD model mirrors the rare human XX DSD subtype

The wide range of XX DSD gonadal phenotypes observed in the canine model is strikingly similar to those observed in human families in which both nonsyndromic OT-XX DSD and T-XX DSD occur [20–27]. The canine XX DSD model closely resembles the nonsyndromic human disorder in expressing both phenotypes within one family and the chromosomal location of the associated insertion. Although the cause in humans is unknown, this type of XX DSD has been mapped to a large region of chr17 (69100001–76800000; OMIM 278850). The XX DSD associated guanine insertion we have identified lies within the canine ortholog for this region.

While rodent models have been useful, they do not have the same range of phenotypic variation observed in these human families. Furthermore, exploration of epigenetic mechanisms in most models has been uncommon. The XX DSD gonadal phenotypes observed in the canine model resemble human patients with *RSPO1* variants, where the *RSPO1* null is associated with testes, and *RSPO1* deficiency is associated with ovotestes. However, *Rspo1* knockout mice develop only ovotestes [5]. In *Rspo1/Sox9* double knockout mice, ovotestes develop in XX mice and other genes in the *SOX* family are thought to induce testis cells [8]. However, in that model, XY mice develop hypoplastic testes, whereas XY siblings of human XX DSD patients are normal, as are XY males in the canine model. Therefore, none of these rodent models mirrors this subtype of human XX DSD.

XX DSD patients with CNVs near *SOX9* are also relevant for comparison, and such syndromic and nonsyndromic patients were recently reviewed [75]. To summarize, the CNV identified are presumed to alter testis-specific regulatory regions of *SOX9* to cause XX DSD. Recent studies refined this region from 42 to 24kb [76,77]. In one of these patients, epigenetic differences were proposed to explain phenotypic expression in the XX DSD patient and absence of expression in the paternal grandmother having the same duplication [74]. Specifically, differences in chromatin signature and methylation patterns identified in adult fibroblast cultures suggested that chromatin modifications may regulate sex determining gene expression. At present, the molecular cause of XX DSD remains unknown in many cases, particularly those patients in which CNVs in the *RevSex* region or *SOX9* duplications have not been identified [78,79]. Also, none of the XX DSD patients from families in which both nonsyndromic testicular and ovotesticular XX DSD have been reported [20–27] has been mapped to the *RevSex* region. The variant identified in this study that is associated with canine XX DSD is relevant to such patients. Examination of the orthologous region in 17q24, surrounding and containing *BTBD17*, is warranted. In addition to further patient studies, canine studies should be useful to identify differences in gene expression and epigenetic marks in embryonic gonads instead of, or in addition to, adult fibroblasts. Such studies could define the steps in molecular pathogenesis, including the role of epigenetic factors in phenotypic penetrance, and the cause of ovarian pathway suppression in developing gonads.

## Materials and methods

### Animals

All research in this study was conducted according to NIH guidelines for the Care of Vertebrate Animals used in Testing, Research, and Training. For pet dogs, the owner’s informed written consent was obtained prior to use of medical information and DNA sample donations for research purposes. Pet owners donated dogs or canine semen used in breeding experiments. All animal care and experimental protocols in this study were approved by the Institutional Animal Care and Use Committee at Cornell University (1989–0068).

#### Diagnostic criteria for canine XX DSD

In previous studies of XX DSD in the model pedigree, all affected dogs had a normal female karyotype (78,XX). Diagnosis in this study was determined by two criteria: 1) presence of seminiferous tubules in either gonad and 2) absence of *SRY* in genomic DNA (below). The proportion of seminiferous tubules in each gonad was estimated from serial histologic sections as: entirely testis (t), ovotestis with greater than one half composed of seminiferous tubules (ovt >0.5t), or ovotestis with less than one half composed of seminiferous tubules (ovt <0.5t, S2 Fig). Neonatal or prepubertal gonads were obtained from most affected dogs, and adult gonads were obtained from breeding stock. For purebred pet dogs, affected dogs were identified by attending veterinarians and diagnosed by the PI (M-W) using the same criteria as in the model pedigree. Where gonadal serial sections from XX DSD pet dogs were unavailable to the PI, gonads were ranked as testis (t), ovotestis (ovt), or ovary (ov) based upon a histopathology report from a veterinary pathologist.

Each animal was genotyped at the *SRY* locus by PCR. Genomic DNA was extracted by the phenol chloroform method [80] or a commercial kit (DNeasy blood and tissue kit, Qiagen). Samples were assayed in triplicate for *SRY* and in duplicate with positive control primers in parallel. Primers were designed from canine *SRY* (GenBank AF107021, [34]): *CfSRY 378F*: CGGAGGAAACGGTAGAGACA; *CfSRY 675R*: GGCTGCAGGTAGCAATTTGT. These flank the HMG box, avoiding hybridization to conserved sequence. Positive control primers were *HMGN2F*: GCCATGTCAGAAACAGTTGG and *HMGN2R*: AAAGGCAGATGCTAACTGAGG. These primer sets produce 298 bp and 425 bp amplicons, respectively. Reactions were run in a standard 4 step thermocycling protocol, reducing annealing temperatures stepwise from 63C to 51C for a total of 37 cycles. The *CfSRY* product was not generated from templates of affected dogs or control females (*SRY-*negative), but was generated from male controls (*SRY-*positive). An *HMGN2* product was generated from all templates.

### To identify variants associated with XX DSD in the canine model

#### GWAS genotyping

Genotyping of 27 dogs (Table 1) was performed using the Affymetrix version 2 canine SNP array (www.broadinstitute.org/mammals/dog/caninearrayfaq.html) and processed with the GeneChip Mapping 250K Sty Assay protocol (Affymetrix Inc., Santa Clara, CA). Genotypes were generated from batched sets of the Affymetrix probe result files (CEL) and were analyzed with canine genotyping software [81]. To identify suggestive variants for further analysis, we analyzed the genome-wide set of genotypes using the GMMAT software [82] to account for confounding effects of genetic relatedness and breed structure among samples, where the random effects matrix was constructed in GEMMA [83] and five Principal Components (PCs) calculated from the genotype matrix filtered for MAF <0.01 and thinned for every 100^th^ SNP were included as fixed effects.

**Further associated variant discovery in the GWAS region by genomic tiling array for SNP genotyping.** DNA samples from 5 dogs were screened with a custom canine genomic tiling array (385K Nimblegen Sequence Capture System, Roche Diagnostic Corp., Indianapolis, IN) composed of 60 bp repeat masked probes overlapping by 3–5 bp in a 2MB region (2604935–3349954 and 5922063–7019389; CanFam3, Fig 2). Coverage for target bases in this design was 97%. Five libraries were prepared from DNA eluted from the array (DNA library preparation kit, Illumina, Inc., San Diego, CA) with approximately 100 fold amplification, judged by quantitative PCR. Libraries were directly sequenced, producing 86 nt reads (GAII, Illumina, BRC Genomics Facility, Cornell University). Read quality was checked (FASTX Toolkit), identifying 83 nt quality reads of single end sequence. Reads were aligned to the reference genome (CanFam2) using default parameters (Novoalign V2.05.04). After conversion to BAM format (Samtools), SNPs and Indels in aligned reads were identified using the Genome Analysis Toolkit (GATK) and genome browsers (IGV, USCS). A total of 8,300 SNPs and 2,182 Indels were selected for further evaluation. These were filtered, eliminating noninformative markers and those in repeats, and retaining those in conserved regions (PhastCons, genome.ucsc.edu ). From these, 5 SNP haplotype blocks of homozygous alternate SNP alleles (*Regions 1–5*) were identified that were the same in ACS1, ACS2 and XX DSD in the model pedigree, but different from those in controls and the reference genome. This provided 732 SNPs/Indels for further homozygosity mapping in SNP haplotype *Regions 1–5*. Of those, 108 loci were selected for genotyping based on the P-value in GWAS, high conservation (PhastCons, genome.ucsc.edu ), or low AF (<0.15) in village dog populations [84]. Sanger sequencing of PCR products was used to genotype 28 affected dogs in the XX DSD pedigree, unrelated affected dogs (8 ACS, 3 ECS, and 29 from 19 other breeds), and 7 female unaffected controls (6 BGL, A168).

**Sanger sequencing of PCR products.** For SNP genotyping, primers flanking the nucleotide of interest were designed from CanFam3.1 (genome.uscs.edu) using Primer3 software (frodo.wi.mit.edu) and *in silico* PCR (genome.uscs.edu). PCR was conducted using the 4 step thermocycling protocol, as for *SRY* PCR (above). PCR products were gel purified (QiaQuick gel extraction kit, Qiagen, Valencia, CA) and Sanger sequenced (ABI 3730xl capillary electrophoresis, BRC Genomics Facility, Cornell University). Sequence texts were evaluated with Clustal software (ebi.ac.uk/Tools/msa/clustalo/) and alleles were verified in electropherograms.

#### CNV detection and SNP genotyping: *Region 3*, canine *RevSex* ortholog, the region near CFA9:6048201, and the *SOX9* coding region

**MLPA for CNV detection in *Region 3* haplotype and the canine *RevSex* ortholog.** MLPA probes were designed according to manufacturer’s directions (Version 12, www.mrc-holland.com) using the Oligo and Peptide design Tool (sigmaaldrich.com). For primer design, conserved sequence between human *RevSex* and the canine ortholog was chosen by using PhastCons, 100 vertebrates basewise conservation by PhyloP, Multiz Alignments of 100 vertebrates, and BLAT functions (genome.ucsc.edu ). Final probe mixes included control probes on other autosomes and 10 test probes in *Region 3*, or 7 probes in the canine *RevSex* ortholog (listed in S2 Table). DNA was extracted from blood or tissue samples using a commercial kit (DNeasy blood and tissue kit, Qiagen) and assayed according to manufacturer’s directions (one-tube protocol, June 2011 version, MDP-v002, MRC-Holland, The Netherlands). Reaction products were submitted for fragment analysis (Big Dye Terminator capillary electrophoresis, ABI 3730, Pathology Department, University of Melbourne Medical School, Melbourne, Australia). Dogs tested in *Region 3* and the canine *RevSex* ortholog included both founder sires, proven carrier females, and XX DSD from the model pedigree, as well as XX DSD pet dogs unrelated to the model pedigree and female and male controls. Affected dogs tested in both regions included T-XX DSD and OT-XX DSD phenotypes (listed in S3 Table).

**SNP genotyping in the canine *RevSex* ortholog by Sanger sequencing.** The canine *RevSex* ortholog (CFA9:17567133–17643805; CanFam3) was identified using the minimum human *RevSex* region shared by patients DSD2 and DSD3 [13] and LiftOver function (genome.ucsc.edu). Primer design and PCR were conducted as for *SRY* PCR and Sanger sequencing (above). Genomic DNA was assayed from 5 dogs (ACS2, C3481, C3023, 2 BGL controls, Table 1) and XX DSD pet dogs from breeds unrelated to the model pedigree.

**ddPCR to identify CNVs near CFA9:6048201 and within the *SOX9* coding region.** Droplet Digital PCR (Bio-Rad Laboratories, Inc., Cambridge, MA) was used to test 5 XX DSD, ACS1 and ACS2 from the model pedigree, and a female control (A168). DNA was BstEII digested prior to PCR (New England BioLabs, Inc., Ipswich, MA). A control and 5 test sets of ddPCR primers and probes (listed in S7 Table) were designed according to manufacturer’s recommendations (Bulletin 6407 Rev A, Bio-Rad Laboratories, Inc.) using public websites (idt.com, sigmaaldrich.com, appliedbiosystems.com). Sequence immediately flanking the 6048201 insertion did not meet probe design standards due to high GC content. The *SOX9* probe binds to a portion of the HMG box in exon 2. Primers and double labeled probes (5’FAM, 3’BHQ1) were manufactured by Eurofinns (Louisville, KY) and Integrated DNA Technologies (Coralville, Iowa), respectively. The ddPCR was conducted according to manufacturer’s recommendations (Bio-Rad Laboratories, Inc., Hercules, CA), using the QX100 Droplet Digital PCR system (BRC Genomics Facility, Cornell University). Briefly, reactions (20ul) containing digested DNA (60 ng), 0.9uM of each primer, 0.25uM of each probe, 1X BioRad Supermix and sterile ultrapure water were assayed in duplicate as absolute quantification experiments (ABS, QuantaSoft software, QX100, Bio-Rad Laboratories).

#### WGS in the canine model to identify additional variants associated with XX DSD

DNA was extracted from blood or tissue samples using a commercial kit (DNeasy blood and tissue kit, Qiagen, Valencia, CA) and evaluated by Bioanalyzer (Agilent Technologies, Santa Clara, CA). A barcoded DNA library for each dog was prepared (TruSeq kit, Illumina), then 2 libraries/lane were sequenced (2x150 bp sequencing, HiSeq Rapid Run, Illumina, BRC Genomics Facility, Cornell University). Using vcftools, we identified all variant SNP/Indel alleles matching the predicted segregation pattern in the 4 dogs.

**Fine mapping by custom array including the WGS discovered variants to identify an XX DSD associated variant in purebred pet dogs.** Affected Pets and Control Pets 1 and 2 (Table 2) were genotyped with a custom designed SNP array of 75 test variants selected from the 244 loci in the WGS region and 9 control SNPs. The array manufacturer (Agena Bioscience, San Diego, CA) designed allele-specific probes at each locus, using sequence provided by investigators. DNA (500ng) from 189 dogs was sheared and size selected, then hybridized to arrays under optimal conditions. For quality control, 3 samples from the Affected Pets group were assayed in duplicate. After removal of nonhybridized DNA, fluorescent signals were identified by array reader and aligned to the allele-specific probe location on the array, generating genotype calls at each locus (Agena Bioscience, SanDiego, CA). Genotype associations were analyzed with PLINK (pngu.mgh.harvard.edu/~purcell/plink/). Genotypes at probes excluded for low call rate were obtained by Sanger sequencing of PCR products, as above.

**Variant analysis in purebred pet dogs to identify an XX DSD associated variant.** DNA samples from 3 purebred pet dog groups unrelated to the XX DSD pedigree were selected for analysis (Table 2). Affected Pets were diagnosed by the PI as above (see Animals). For Control Pets 1 and 2, DNA was purchased from the Cornell Veterinary BioBank, an archive of DNA from pet dogs examined at the Cornell University Hospital for Animals, College of Veterinary Medicine, Ithaca, NY [85]. Samples were paired to medical information from a standard medical screen, including physical examination of the external genitalia by board certified veterinary specialists. DNA for Control Pets was extracted using commercial reagents (Gentra PureGene Blood Kit, Qiagen, Valencia, CA). DNA for Affected Pets was extracted by the phenol chloroform method [80] or commercial reagents (DNeasy blood and tissue kit, Qiagen) and some were whole genome amplified (REPLI-g Midi kit, Qiagen Inc., Valencia, CA). Breeds by group are listed in S6 Table.

**Genotyping at or near CFA9:6048201.** Dogs in the model pedigree and experimental breedings (Fig 3) were genotyped by sequencing PCR products that included the 6048201 locus (S5 Fig), as in Sanger sequencing (above). Genotypes for pet dogs were confirmed by this method. To identify sequence variations near CFA9:6048201, PCR primers were designed to amplify products (230–992 bp) of contiguous sequence within 6046484–6048494 (CanFam3). A Taq enzyme for GC rich targets was used, according to manufacturer’s recommendations (KOD Xtreme Hot Start, Novagen, Merck KGaA, Darmstadt, Germany). Templates included DNA from XX DSD, ACS1 and ACS2 from the model pedigree, and female controls (beagles and A168).

### Gene expression in canine embryonic gonads

#### Gonad collection for RNA-seq and PRO-seq analyses

Embryos at risk of XX DSD and XY littermate controls were produced by ACS/BGL females bred to ACS2 or ACS/BGL XY males in the study pedigree. Because the causative mutation is unknown, and there are no ACS lines certified free of XX DSD, age-matched controls were collected from BGL parents of the same genetic background as those bred into the study pedigree. The day of embryo collection was timed from d0, the day of the preovulatory serum luteinizing hormone (LH) surge and concomitant rise in serum progesterone (P4) concentration in the dam [86]. By using serial preovulatory P4 concentrations alone to identify d0, unassisted parturition occurs at d65 (+/- 2). This method is 90% accurate in predicting gestation length in all breeds, regardless of litter size and body weight of the dam [87,88] and has been used to time ovulation and gestation for *in vitro* fertilization [89], embryo transfer [90], and previous gonadal expression studies [49,91]. Embryos were collected by hysterotomy under general anesthesia in aseptic conditions and maintained in RNAse free phosphate buffered saline (PBS, 4C) throughout microdissection. Crown rump length was measured and photographs were taken during microdissection (DP11 digital camera, Olympus America, Melville, NY). Samples for DNA, RNA, or chromatin, cataloged by embryo identification number, were flash frozen and stored in liquid nitrogen. Each embryo was developmentally staged according to photographed external morphology and genotyped by PCR at *SRY* and CFA9:6048201 loci, as above (Animals).

#### RNA-seq library preparation and analysis of gene expression in embryonic gonads

For library preparation, total RNA from paired gonads of each canine embryo was extracted using commercial reagents (RNeasy Plus Mini Kit, Qiagen, Hilden, Germany). However, d34 gonads were too small to provide sufficient RNA, therefore gonads from these were pooled into two d34 control groups according to *SRY* assay result (+/-), and RNA was extracted from each pool. For all others (d35-44), barcoded cDNA libraries were prepared from total RNA of each gonad pair (TruSeq RNA Sample Prep Kit v2, Illumina, San Diego, CA), then pooled for sequencing according to group (XX DSD at risk or control). Libraries were sequenced with 75-100nt single end reads (NextSeq500 or HiSeq 2000/2500, Illumina, BRC Genomics Facility, Cornell University). Sequence output was sorted by barcode to generate one transcriptome per gonad pair. An overall average of 40M reads was generated per library. Prior to analysis, reads were trimmed with cutadapt v1.8 to remove low quality and adaptor sequences from the 3’ ends, and filtered to remove trimmed reads <20nt (parameters: -m 20 -q 20 -a AGATCGGAAGAGCACACGTCTGAACTCCAGTC—match-read-wildcards). Reads were mapped to the CanFam3 reference genome with tophat v2.0, using the Ensembl annotated transcriptome. Overall, an average of 30M reads per library was mapped to the reference genome.

Cufflinks v2.2 (cuffnorm/cuffdiff) [92] was used to generate gene expression values in FPKM units for all annotated genes, and to perform statistical analysis of differential gene expression between samples from XX DSD gonads at risk and XX age-matched controls and at gestational age groups d37-39 and d42-44. Because FPKM units are normalized to the length of the transcript, these units are proportional to gene expression levels and can be compared across genes and between samples. The cuffdiff software uses q-value cutoff of 0.05 to distinguish genes with significantly different expression between groups. A more stringent list of differentially expressed genes was used for each age group by requiring a minimum 2-fold change between XX DSD gonads at risk and XX age-matched control groups [abs(log2FC)≥1] and at least one group to have an average of FPKM ≥4.

The Jmp Pro 11 software (SAS, Cary, NC) was used for principal components analysis (PCA) and hierarchical clustering. PantherDB (pantherdb.org) was used for statistical over representation tests for GO terms [93]. PCA comparing differential expression in gonads of all groups and ages confirmed expression clustered according to *SRY* assay result (+/-), age, and XX DSD at risk or control status. Because gene expression in XX DSD gonads at risk was more similar to that of XX age-matched controls than XY littermates, we used PCA to compare expression in XX DSD at risk and XX age-matched controls at the d37-39 and d42-44 age groups. Although expression in most samples clustered appropriately by age and status, four samples in the d37-39 group clustered with XX age-matched controls of the older group. Because developmental criteria were the same for d37 controls (A1097, A1098) and XX DSD C3512 and C3515, the latter were analyzed with the d37-39 group. Developmental criteria indicated XX DSD C3694 and C3696 and XY littermate C3691 were >d39 but <d42 in comparison to controls, and these were analyzed with the d42-44 group.

#### PRO-seq library preparation and analysis of gene expression in embryonic gonads

Canine embryonic gonad pairs were assigned to test or control groups according to *SRY* assay result (+/-) and genotype at the insertion locus (Table 6). Gonad pairs were homogenized by mechanical disruption (Pestle Motor Mixer) in NUN buffer (20 mM HEPES, pH 7.5, 7.5 mM MgCl2, 0.2 mM EDTA, 0.3 M NaCl, 1M Urea, 1% NP-40, 1 mM DTT, 20 units/ml SuperaseIn RNase Inhibitor, 1X Protease Inhibitor Cocktail). Chromatin was pelleted by centrifugation (30 mins, 4C) and re-suspended in storage buffer (50mM Tris-HCl, pH 8, 25% glycerol, 5mM MgAc2, 0.1mM EDTA, 5mM DTT, 40 U/ml SUPERase In RNase Inhibitor) by sonication (10 mins, Duty cycle: 30 sec on/30 sec off) in a Bioruptor (Diagenode Inc., Denville, NJ).

PRO-seq library preparation was completed as previously described [47,94] with three minor modifications. 1) The run on reaction was completed on isolated chromatin. 2) Only two biotinylated (U and C) and two non-biotinylated (G and A) nucleotides were used, providing high resolution for the RNA polymerase active site location at an economical cost per library. 3) Adapters were modified to contain a 6 nucleotide molecule-specific barcode ID at the 5’ end of each sequenced RNA. Samples were sequenced using a single lane (Illumina NextSeq500, BRC Genomics Facility, Cornell University).

Data were processed and samples aligned using our publicly available alignment pipeline (github.com/Danko-Lab/utils/tree/master/proseq, commit: 425de21). Briefly, after removing reads that failed Illumina quality filters, duplicate reads were removed and the 6 bp molecule-specific ID was trimmed from the 5’ end of each read using prinseq lite [95]. Adapters were trimmed using cutadapt with a 10% error rate [96]. Finally, reads were mapped with BWA [97] to a reference genome comprised of CanFam3.1 and a single copy of the Pol I ribosomal RNA (GenBank U13369.1). Samples were visualized using custom track hubs on the WashU epigenome browser [98]. The dREG software program was used to identify the location of promoters and active enhancers in combined PRO-seq data [99], combining male and female samples to improve statistical power for identifying transcriptional regulatory elements.

## Supporting information

S1 Fig Canine model pedigree in which prevalence and severity of XX DSD decreased as beagle background increased. Females are indicated by open circles, XX DSD by filled circles, and XY males by squares. The filled square represents founder sire ACS1. Each symbol represents one dog, except those containing a number, which indicates the number of dogs represented by that symbol. Beagle (BGL) females introduced later into the pedigree (N = 4) have numbers beginning with A.
(TIF)

S2 Fig Examples of gonadal histology identified in canine XX DSD and controls. (ZIP)

S3 Fig GWAS Quantile-Quantile Plot of the p-values used to assess GWAS model fit and significance. The plot shows that most of the genotypes follow the expected null distribution and marginally significant p-values at the extreme (corresponding to a region of CFA9).
(TIF)

S4 Fig Distribution of 754 stringent-DE genes across 10MB intervals of the CanFam3 genome. (PDF)

S5 Fig Genomic sequence flanking the insertion associated with XX DSD (CFA9:6048201–6048202; CanFam3.1). The guanine insertion is in bold type and enclosed by square brackets. Curved brackets in bold type enclose the SNP array probe sequence for this locus. PCR primers used to genotype this locus (6048201_F1 and 6048201_R1) are underlined.
(TIF)

S1 Appendix Chi square analysis of the proportion of affected offspring in the early and later canine XX DSD model pedigree. (TIF)

S2 Appendix Chi square analysis of the proportion of mild and severe phenotypes in affected offspring in the early and later canine XX DSD model pedigree. (TIF)

S3 Appendix GWAS phenotype file: pheno.txt. (TXT)

S4 Appendix GWAS PLINK file: plink.ped. (PED)

S5 Appendix GWAS PLINK file: plink.map. (MAP)

S1 Table Results of the GWAS logistic mixed model analysis. (TXT)

S2 Table MLPA probes tested in *Region 3* haplotype block and the canine *RevSex* ortholog on CFA9. (XLSX)

S3 Table Dogs tested by MLPA to screen for CNVs in haplotype *Region 3* and the canine *RevSex* ortholog. (XLSX)

S4 Table List of 244 CFA9 variants identified by WGS of 3 dogs from the XX DSD model pedigree and 1 female control. (XLSX)

S5 Table Variant and control loci used in the custom canine array of WGS variants to identify those associated with XX DSD in Affected Pets compared to the Control Pets 1 and 2 groups. (XLSX)

S6 Table Genotypes at the CFA9:6048201 locus for all pet dogs tested in the custom canine array of WGS variants. (XLSX)

S7 Table Droplet Digital PCR probes used near the CFA9:6048201 locus and in the *SOX9* coding region to screen for CNV in XX DSD from the model pedigree and controls. (XLSX)

S8 Table Potential transcription factor binding sites introduced by the guanine insertion at CFA9:6048201. (XLSX)
