# Supplementary figures and images for: XX Disorder of Sex Development is associated with an insertion on chromosome 9 and downregulation of RSPO1 in dogs (Canis lupus familiaris)
Source: PLoS One. 2017 Oct 20;12(10):e0186331. doi: 10.1371/journal.pone.0186331 (PMC5650465; doi:10.1371/journal.pone.0186331)

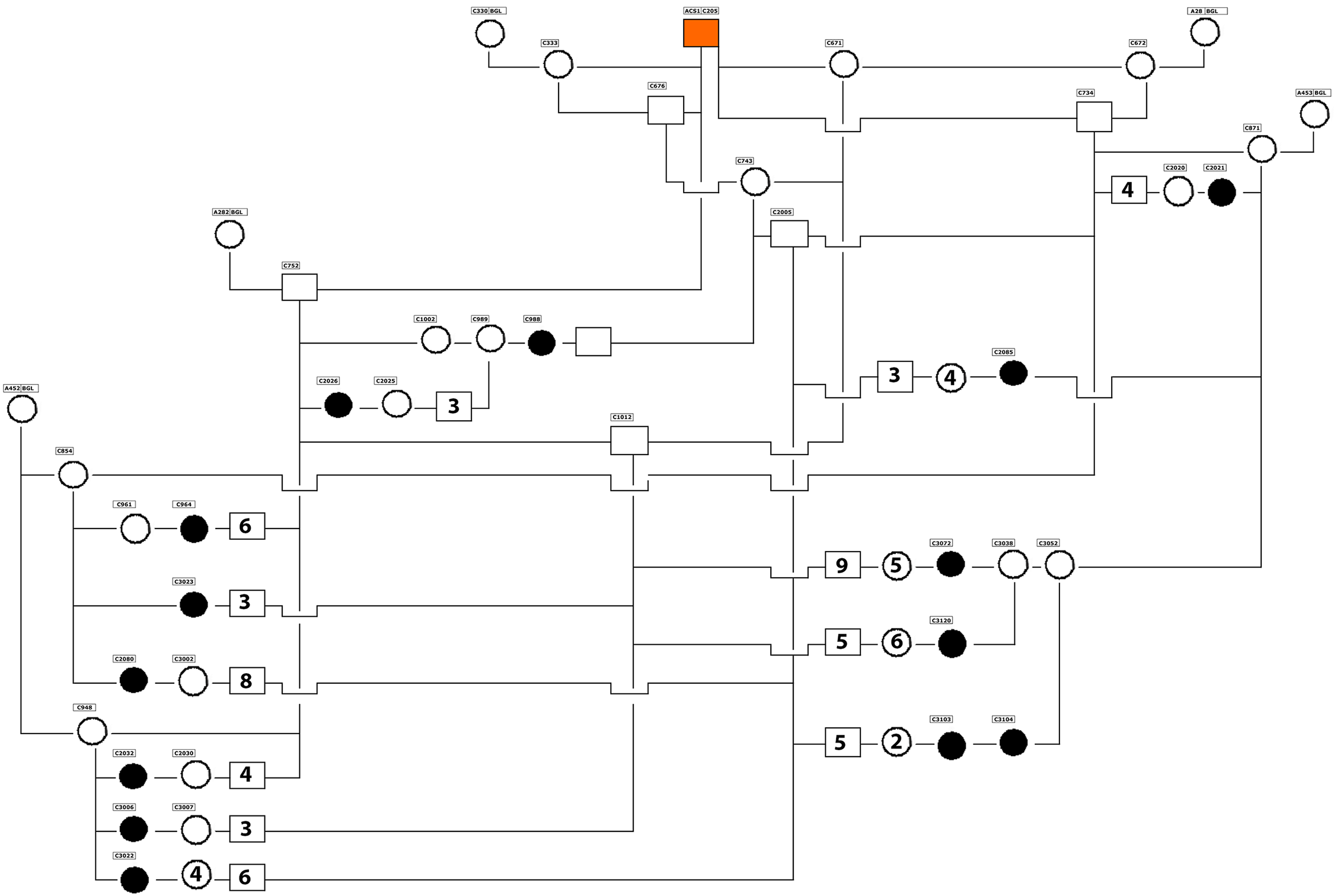

Supplement: S1 Fig — Females are indicated by open circles, XX DSD by filled circles, and XY males by squares. The filled square represents founder sire ACS1. Each symbol represents one dog, except those containing a number, which indicates the number of dogs represented by that symbol. Beagle (BGL) females introduced later into the pedigree (N = 4) have numbers beginning with A. (TIF) [file pone.0186331.s001.tif]

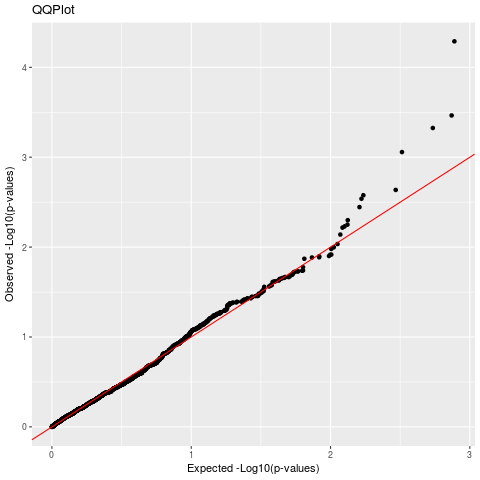

Supplement: S3 Fig — The plot shows that most of the genotypes follow the expected null distribution and marginally significant p-values at the extreme (corresponding to a region of CFA9). (TIF) [file pone.0186331.s003.tif]

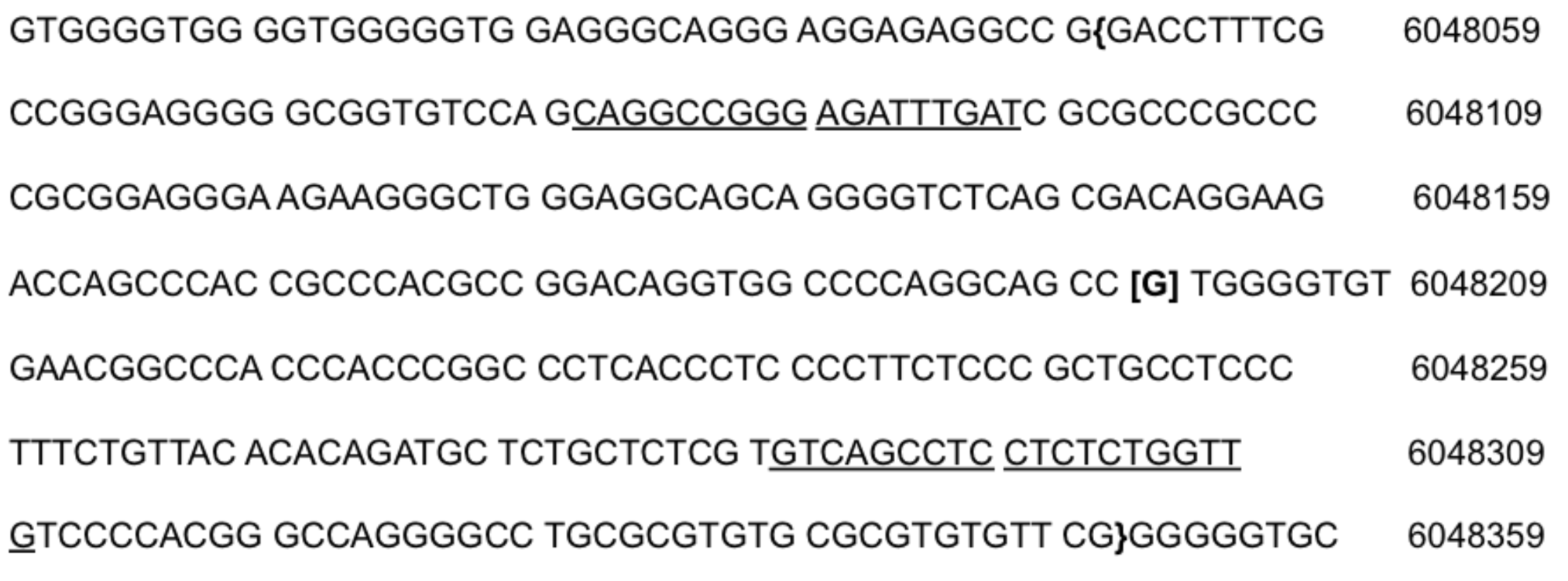

Supplement: S5 Fig — The guanine insertion is in bold type and enclosed by square brackets. Curved brackets in bold type enclose the SNP array probe sequence for this locus. PCR primers used to genotype this locus (6048201_F1 and 6048201_R1) are underlined. (TIF) [file pone.0186331.s005.tif]

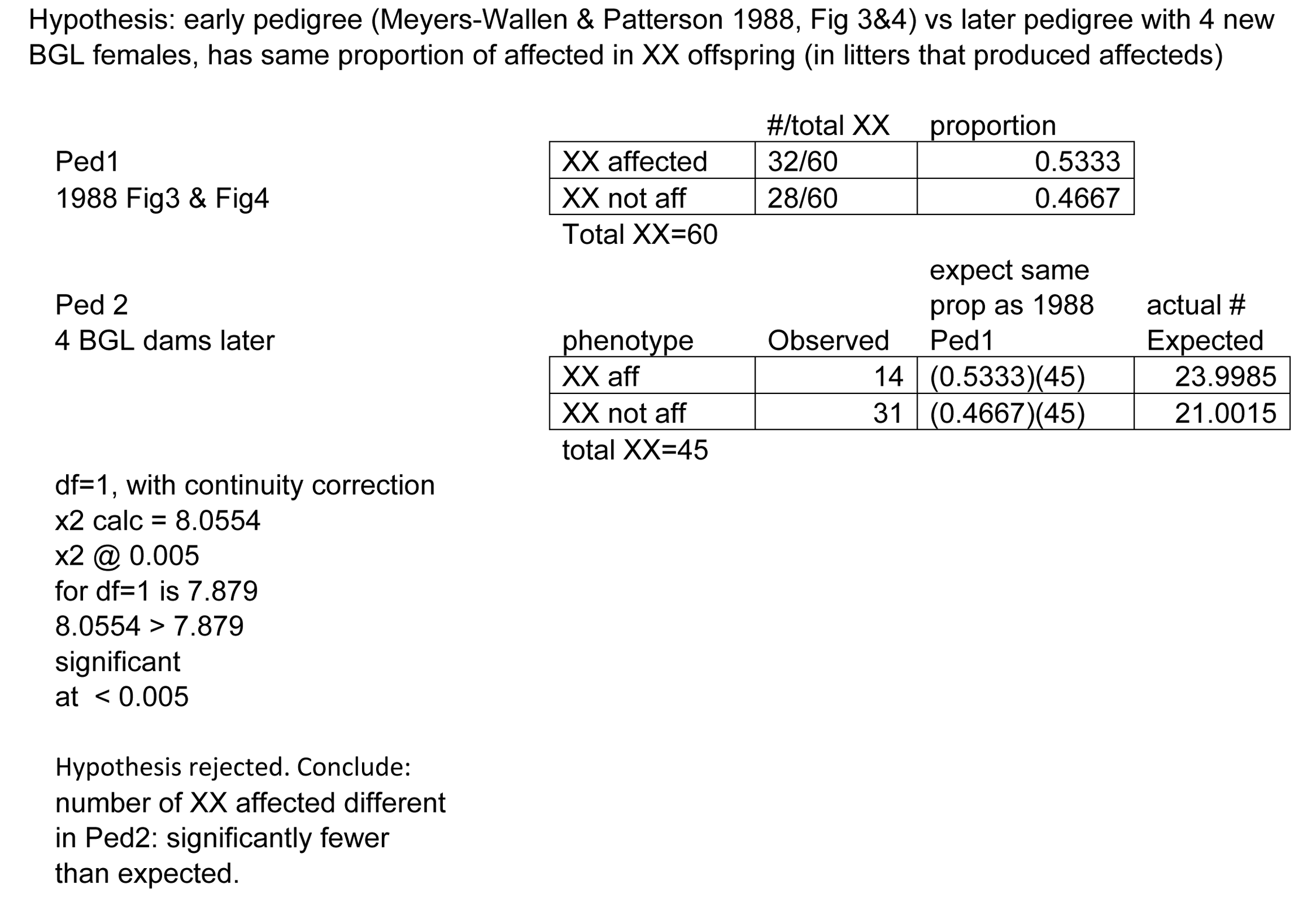

Supplement: S1 Appendix — (TIF) [file pone.0186331.s006.tif]

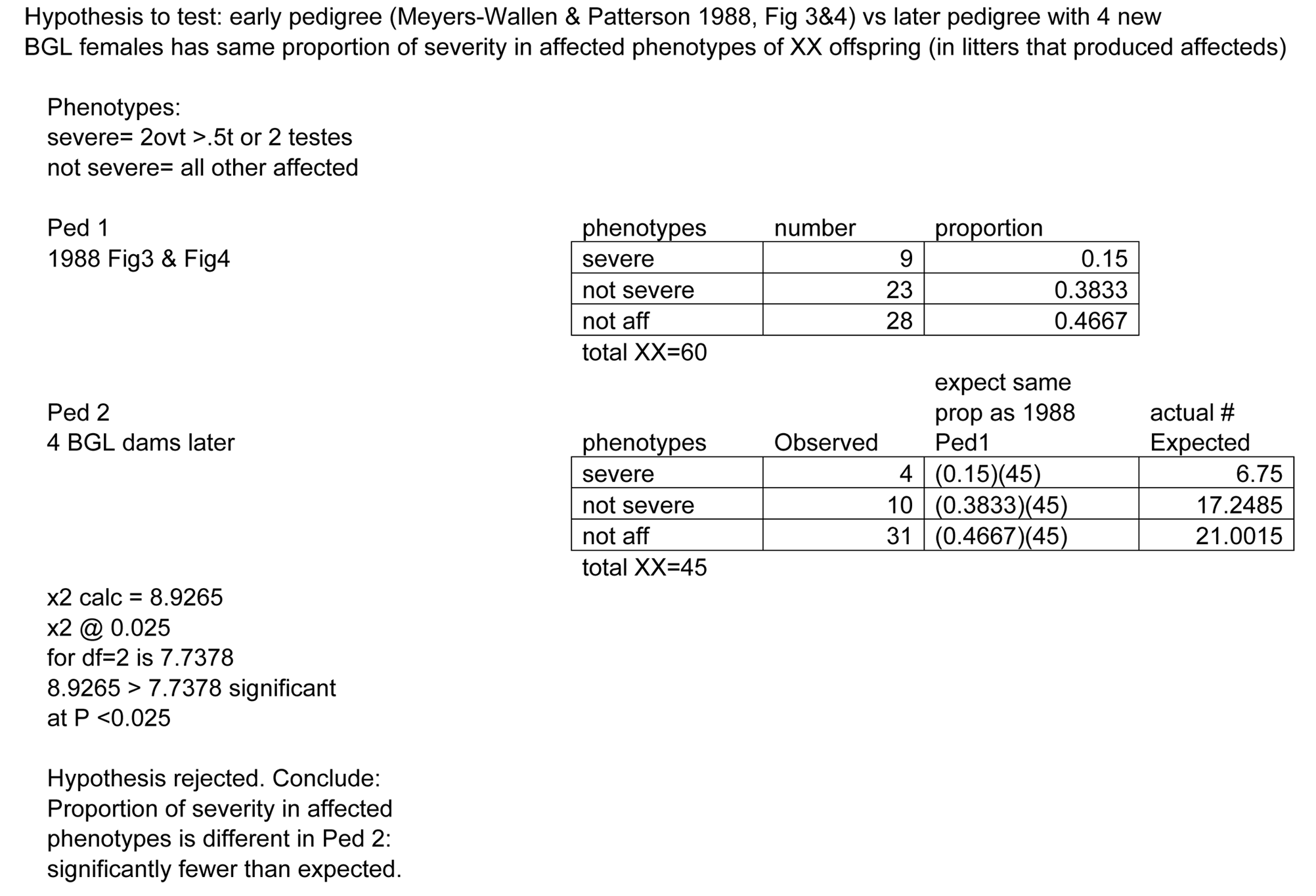

Supplement: S2 Appendix — (TIF) [file pone.0186331.s007.tif]
