# Supplementary material for: XX Disorder of Sex Development is associated with an insertion on chromosome 9 and downregulation of RSPO1 in dogs (Canis lupus familiaris)
Source: PLoS One. 2017 Oct 20;12(10):e0186331. doi: 10.1371/journal.pone.0186331 (PMC5650465; doi:10.1371/journal.pone.0186331)
Supplement: S2 Fig — (ZIP) [file pone.0186331.s002.zip › S2_Fig_FINAL_10-4-17.pdf]

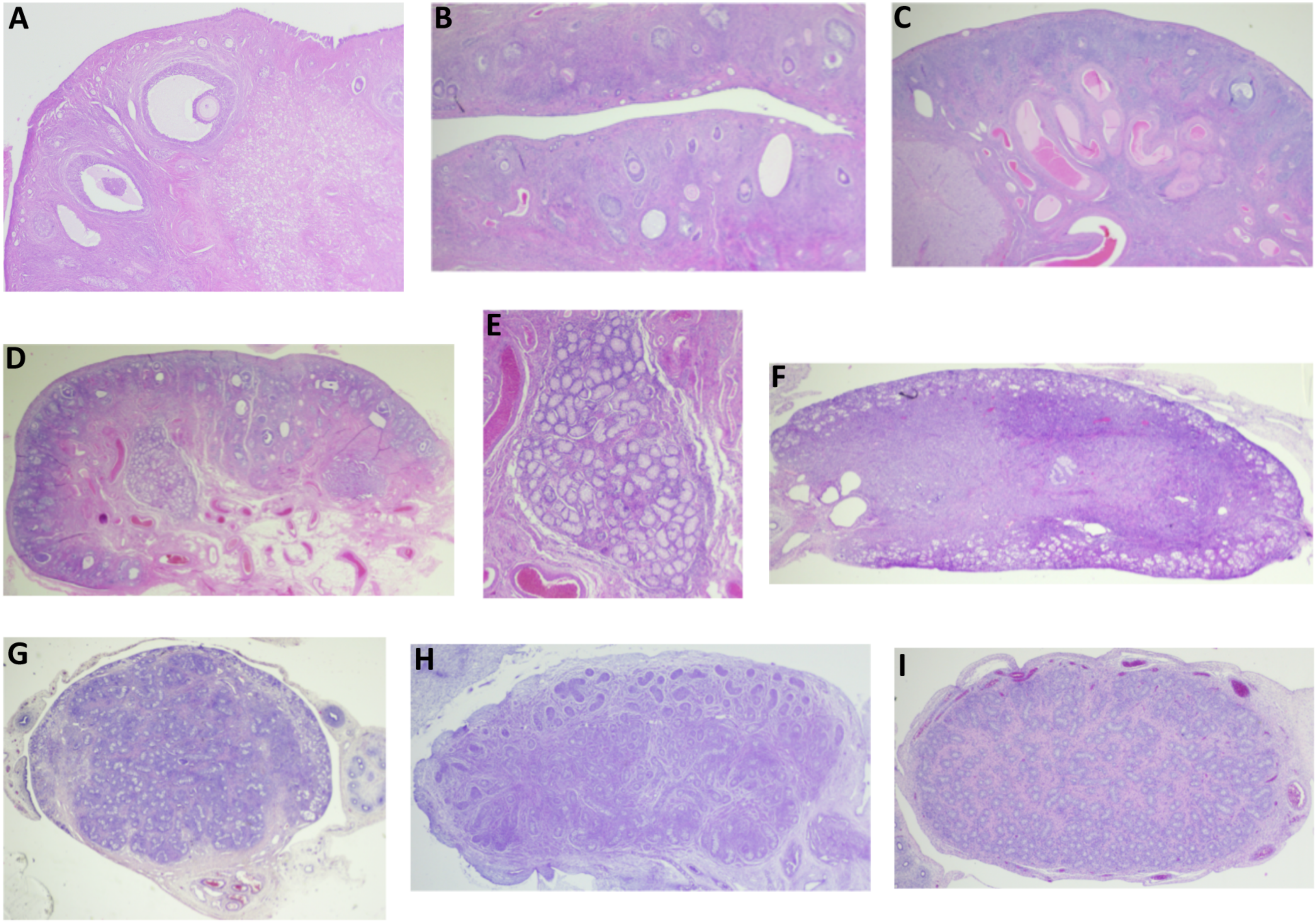

S2 Fig. Adult canine ovaries: A. A888 control female beagle (Table 1), age 4 yrs 5 mo. Oocytes lie within follicles of various stages in the cortex. Beneath the largest follicle on the right is a regressing corpus luteum. **B.** C3052 female carrier, age 8 yrs 9 mo. Follicles of various stages are in the cortex, with germinal epithelium at the cortical surface. **C.** C3466 female carrier (Table 1), age 4 yrs 10 mo. Follicles of various stages are in the cortex, with germinal epithelium at the cortical surface. On the left, a large corpus luteum is present in the cortex. **Adult canine OT-XX DSD, Ovotestis <.5 testis: D.** C3468 OT-XX DSD (Table 1), age 1 yr 7 mo. A large group of seminiferous tubules is present in the medulla, but comprises less than half the entire gonad. Numerous follicles of various stages are in the cortex. **E.** C3468, same ovotestis as D, with the group of seminiferous tubules at higher magnification showing absence of germ cells. **Neonatal canine OT-XX DSD, Ovotestis: F.** C3497, Ovotestis <.5 testis, age 10 days. A small group of seminiferous tubules is present in the center medulla. Numerous small follicles are present in the cortex. **G.** C783 (Table 1), Ovotestis >.5 testis, age 14 days. Several groups of seminiferous tubules are present in the medulla, with numerous small follicles in the cortex. **Neonatal canine T-XX DSD Testis: H**. C2080 (Table 1), age 1 day. Multiple seminiferous tubules are present throughout the gonad and the tunica albuginea surrounds the cortical surface. **Neonatal canine XY Testis: I.** C3714, age 1 day. Seminiferous tubules with intervening interstitial cells are present and the tunica albuginea surrounds the cortical surface.
