# Supplementary material for: XX Disorder of Sex Development is associated with an insertion on chromosome 9 and downregulation of RSPO1 in dogs (Canis lupus familiaris)
Source: PLoS One. 2017 Oct 20;12(10):e0186331. doi: 10.1371/journal.pone.0186331 (PMC5650465; doi:10.1371/journal.pone.0186331)
Supplement: S4 Fig — (PDF) [file pone.0186331.s004.pdf]

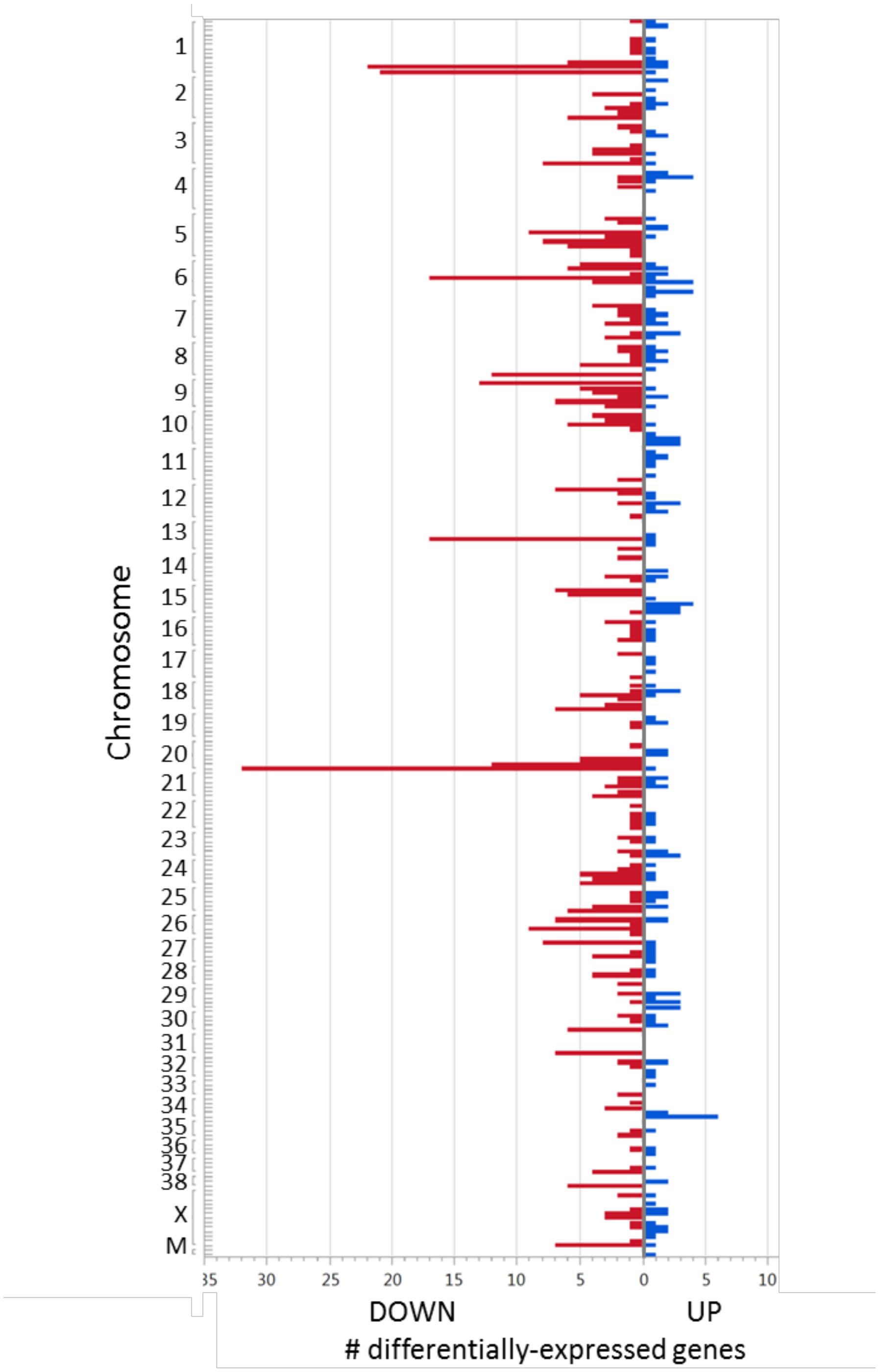

Distribution of 754 stringent-DE genes across 10Mb intervals of the CanFam3 genome.
The majority of these differentially-expressed genes have lower expression in the XX DSD cohort (red bars; 539=71%). Genomic intervals of 10Kb with at least 10 downregulated genes include Chr1: 100-110Mb (22 genes) and 110-120Mb (21 genes), Chr6: 30-40Mb (17 genes), Chr8:60-73Mb (12 genes), Chr9: 0-10Mb (13 genes; this region includes the GWAS and WGS intervals), Chr13: 30-40Mb (17 genes), Chr20:40-50Mb (12 genes) and 50-60Mb (32 genes).
